# A conserved domain targets exported PHISTb family proteins to the periphery of *Plasmodium* infected erythrocytes

**DOI:** 10.1016/j.molbiopara.2014.07.011

**Published:** 2014-08

**Authors:** Sarah J. Tarr, Robert W. Moon, Iris Hardege, Andrew R. Osborne

**Affiliations:** a Institute of Structural and Molecular Biology, Division of Biosciences, Birkbeck and University College London, London, UK; b Division of Parasitology, MRC National Institute for Medical Research, London, UK

**Keywords:** PHIST, PRESAN, *Plasmodium*, Cytoskeleton, Protein export, Malaria

## Abstract

During blood-stage infection, malaria parasites export numerous proteins to the host erythrocyte. The Poly-Helical Interspersed Sub-Telomeric (PHIST) proteins are an exported family that share a common ‘PRESAN’ domain, and include numerous members in *Plasmodium falciparum*, *Plasmodium vivax* and *Plasmodium knowlesi*. In *P. falciparum*, PHIST proteins have been implicated in protein trafficking and intercellular communication. A number of PHIST proteins are essential for parasite survival. Here, we identify nine members of the PHISTb sub-class of PHIST proteins, including one protein known to be essential for parasite survival, that localise to the erythrocyte periphery. These proteins have solubility characteristics consistent with their association with the erythrocyte cytoskeleton. Together, an extended PRESAN domain, comprising the PRESAN domain and preceding sequence, form a novel targeting-domain that is sufficient to localise a protein to the erythrocyte periphery. We validate the role of this domain in RESA, thus identifying a cytoskeleton-binding domain in RESA that functions independently of its known spectrin-binding domain. Our data suggest that some PHISTb proteins may act as cross-linkers of the erythrocyte cytoskeleton. We also show for the first time that peripherally-localised PHISTb proteins are encoded in genomes of *P. knowlesi* and *vivax* indicating a conserved role for the extended PRESAN domain of these proteins in targeting to the erythrocyte periphery.

## Highlights

• Multiple *P. falciparum* PHISTb proteins localise to the erythrocyte periphery.
• Solubility profiling indicates that these proteins associate with the red cell cytoskeleton.
• The PRESAN domain and a preceding N-terminal sequence is a novel targeting domain.
• A protein targeted to the red cell periphery is essential for parasite survival.
• *P. knowlesi* and *P. vivax* homologous domains also confer similar localisation.

## Graphical abstract

The extended PRESAN domain is a targeting domain used by multiple *Plasmodium* species to target PHISTb proteins to the cytoskeleton/plasma membrane of infected cells.

**Figure:**
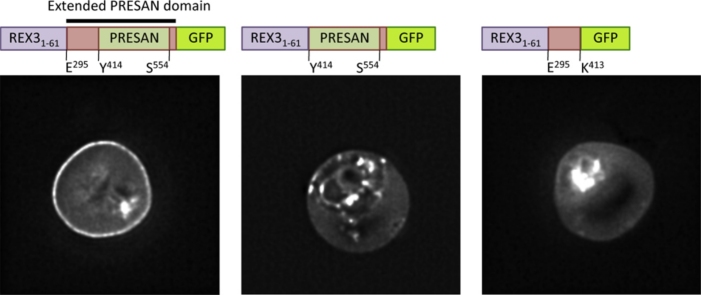

## 1 Introduction

Malaria is caused by eukaryotic parasites of the genus *Plasmodium*, of which five species are known to infect humans. There were an estimated 627 000 deaths from malaria in 2012 [1]; the majority occurred in children in sub-Saharan Africa as a result of infection with *Plasmodium falciparum* which causes the most deadly form of the disease. Infections with other human malaria species contribute significantly to the global impact of malaria; up to 50% of malaria cases outside sub-saharan Africa are attributed to infection with *Plasmodium vivax* [1], which is increasingly recognised as a cause of severe malaria and death. Malaria caused by *Plasmodium knowlesi* is primarily zoonotic and often misdiagnosed. While infection with *P. knowlesi* can cause severe and fatal disease [2], the contribution of *P. knowlesi* to the global malaria burden is only beginning to be understood as a consequence of recently developed diagnostic strategies allowing its accurate diagnosis [2].

Symptoms of malaria occur when the parasite resides and replicates within host erythrocytes (although *P. vivax* preferentially invades reticulocytes). Within the erythrocyte, the parasite is contained within a vacuole, referred to as the parasitophorous vacuole (PV). During the intraerythrocytic cycle, *Plasmodium* parasites export many proteins across the PV membrane and into the host erythrocyte. Many exported proteins are directed for export by a host-targeting (HT) motif (also referred to as a PEXEL) [3], [4], the presence of which can be used to identify putative exported proteins. During synthesis, most proteins destined for export are initially targeting to the parasite endoplasmic reticulum by an N-terminal transmembrane domain (or recessed signal sequence) [5], [6]. The HT motif lies downstream of the N-terminal transmembrane domain [3], [4], [7]. Within the ER lumen, the HT motif is proteolytically cleaved after the Leu residue [8], [9] by the ER-resident protease Plasmepsin V [5], [10], releasing a C-terminal fragment with a new N-terminal sequence that starts xE/D/Q, which is N-acetylated [8]. The new N-terminus alone is sufficient to mediate protein export [11], [12]. The cleaved, N-acetylated proteins are trafficked to the PV where they are unfolded and translocated across the PV membrane by the PTEX complex [13], [14]. An important subset of proteins lacking HT motifs is also exported into the host cell [15].

Many exported proteins, which alter the properties of infected cells, are essential for parasite survival [16] and virulence [17], [18] and hence contribute to the pathogenesis of malaria [19], [20]. Infected erythrocytes exhibit increased nutrient uptake, increased rigidity and the ability to cytoadhere to microvasculature [21]. Much of the increased rigidity of infected erythrocytes is attributed to the association of KAHRP with the cytoskeleton [22]. KAHRP also plays a key role in the assembly of cytoadherence-associated knob structures on the plasma membrane of infected cells [23]. KAHRP binds PfEMP1-ATS and Spectrin-actin-4.1 complex thus linking PfEMP1 to the cytoskeleton [24] (see also [25]). Parasites lacking KAHRP have a knob-less phenotype and fail to generate a virulent infection in primates [26]. Other cytoskeleton binding proteins have been identified in *Plasmodium*. The Mature-parasite-infected Erythrocyte Surface Antigen (MESA) interacts with Band 4.1 in the erythrocyte cytoskeleton [27], [28], [29]. A similar binding region is found in a number of other *Plasmodium* exported proteins [30].

However, the majority of *Plasmodium* parasite exported proteins have not been characterised. One family that is expanded in human-infective *Plasmodium* species is the Poly-Helical Interspersed Sub-Telomeric (PHIST) protein family. The PHIST family was identified by two groups independently and is also known as the PRESAN family [31], [32]. In *P. falciparum*, the PHIST family contains approximately 74 members. Whilst PHIST family proteins are diverse and often multi-domain proteins, they share a single (or occasionally two) conserved, four-helical region of approximately 150 amino acids in length named the PHIST or PRESAN domain (Pfam ID: PF09687). PHIST family is further divided into three sub-families, named PHISTa, PHISTb and PHISTc [31]. Henceforth, ‘PHIST’ will be used to describe PHIST/PRESAN family members, while ‘PRESAN’ will be used to describe their common domain. The PHIST family proteins are also found in other *Plasmodium* species; notably there are multiple members present rodent malaria parasites but also in both *P. vivax* and *P. knowlesi* [31], [33], [34].

In *P. falciparum* three PHISTb proteins (PF3D7_0401800 (PFD80), PF3D7_0402100 and PF3D7_1149200) are essential for parasite survival in blood stages [16]. The *Plasmodium cynomolgi* protein PHIST/CVC-81_95_, is most closely related to PHISTc proteins [35], localised in Schüffner's dots in the infected erythrocyte, and is also refractory to gene deletion [36]. Some non-essential PHIST family members from *P. falciparum* are involved in functions such as protein trafficking, membrane rigidity and intercellular signalling [16], [37], [38].

Interacting partners have only been identified for a small number of PHIST proteins. The PHISTa protein PF3D7_0402000 is localised to the PV membrane and the PRESAN domain of this protein interacts with the cytoplasmic erythrocyte protein Band 4.1 in a yeast two-hybrid assay [39]. Biochemical analyses have identified an interaction between the PRESAN domain of the PHISTc protein PF3D7_0936800 and the intracellular acidic tail sequence domain of PfEMP1 [25]. Interactions of PHISTb proteins have been identified for regions outside of their PRESAN domains. The PHISTb protein, Ring-infected Erythrocyte Surface Antigen (RESA; PF3D7_0102200) is localised to the erythrocyte membrane [40], [41] and contains a C-terminal spectrin-binding domain [42], [43]. The direct association of RESA with spectrin stabilises the spectrin tetramer and is key to the increased resistance of infected erythrocytes to mechanical stress and thermal damage [43], [44], [45]. PF3D7_0532400 was also recently shown to associate with inside-out vesicles via its C-terminal lysine-rich repeat region, and partially colocalise with KAHRP, suggesting an interaction with the erythrocyte cytoskeleton [46].

Although the virulence-associated phenotypes of malaria parasites involve modification of the host cell plasma membrane and cytoskeleton, our understanding of how proteins are targeted to this region of the infected erythrocyte is incomplete. Here we identify seven members of the PHISTb family in *P. falciparum* that localise to the erythrocyte periphery. The solubility characteristics of these proteins are consistent with their association with the erythrocyte cytoskeleton. Using the PHISTb proteins PFD80 and RESA as model proteins, we show that the PRESAN domain together with a preceding sequence, comprise a novel functional domain that is sufficient to target a protein to the erythrocyte periphery. Furthermore, we show for the first time that PHISTb proteins encoded in the genomes of *P. vivax* and *P. knowlesi* are also peripherally-localised in infected erythrocytes indicating that the extended PRESAN domain found in PHISTb proteins is a conserved, universal domain used to target proteins to the periphery of *Plasmodium* infected erythrocytes.

## 2 Experimental procedures

### 2.1 Plasmids and parasite transfection

DNA encoding *P. falciparum* PHIST proteins was PCR amplified from *P. falciparum* 3D7 genomic DNA and cloned into a *P. falciparum* expression plasmid, in frame with 3′ GFP and StrepII tags. Expression was under the control of pfCAM 5′ and pbDT 3′ regions. DNA fusions of PHIST protein domains with the N-terminal sixty-one residues of PF3D7_0936300 (REX3/PFI1755c) were generated using overlapping PCR.

The sequence for PKH_103230 was PCR amplified from *P. knowlesi* genomic DNA (from strain A1H.1 [47]) and the intron was removed. The gene was cloned in frame, with 3′ GFP and StrepII tags, into the *P. knowlesi* expression vector, PkconGFPep [47]. For expression of PKH_103230 in *P. falciparum*, a plasmid was constructed as described above for expression of *P. falciparum* PHIST proteins. The fragment of the gene PVX_003555 encoding the PRESAN and preceding region (residues 807-end), was amplified from *P. vivax* genomic DNA (from an undefined strain – a gift from Colin Sutherland). This fragment was cloned such that it was fused to residues 1–61 of PF3D7_0936300 (REX3), and also in frame with 3′ GFP and StrepII tags; expression was under the control of pfCAM 5′ and pbDT 3′ regions.

Protein expression plasmids and the pINT No Neo plasmid (derived from pINT [48] but lacking a Neomycin selection gene), encoding the Bxb1 integrase, were co-transfected into *Plasmodium falciparum* strain 3D7attB or Dd2attB parasites [48] as described previously [49] or [47]. Transfected parasites were selected with 2 μg/ml Blasticidin (plasmid integration into the AttB site was not verified). *P. knowlesi* parasites adapted to continuous culture in human erythrocytes were maintained and transfected as described in [47], with the exception that parasites were selected with 300 nM pyrimethamine.

### 2.2 Microscopy

One day after feeding, mixed-stage parasites were imaged by placing a drop of culture material between a microscope slide and coverslip. Phase contrast and fluorescence Z-stack images were acquired using a Zeiss Axiovert 200 M microscope, equipped with a HBO100 lamp and Axiovision software. Fluorescence Z-stacks were deconvolved using Volocity software. Images were processed in ImageJ and single image sections are presented. Fluorescence images were adjusted using automatic brightness and contrast settings.

### 2.3 Protein detection by Western blotting

Expression of GFP-tagged proteins in transfected parasite lines was confirmed by SDS-PAGE and Western blotting of purified schizonts. 2 × 10^6^ schizonts were loaded per lane. Western blots were probed with rabbit anti-GFP antibody (Torrey-Pines). Western blots were imaged using a LI-COR Odyssey imager.

### 2.4 Preparation of erythrocyte ghosts and triton shells

Extraction of PHISTb proteins from erythrocyte ghosts and membrane-depleted triton shells was conducted by sequential extraction of purified schizonts in hypotonic and triton buffers. Schizonts were resuspended in 7.5 mM sodium phosphate, pH 7.4 at 4 °C and centrifuged at 18,500 × *g* for 10 min. The pellets, containing ghosts, and supernatants were separated. Triton shells were prepared by resuspending the ghost pellet in chilled 1% triton X-100 in PBS, followed by centrifugation at 18,500 × *g* for 10 min. Pellets and supernatants were separated.

Prior to SDS-PAGE, pellet fractions were resuspended in the starting volume of buffer. After addition of sample buffer, all samples were heated to 95 °C for 1 min. The equivalent of 2 × 10^6^ schizonts were loaded per lane for separation by SDS-PAGE, followed by Western blotting onto nitrocellulose. Western blots were probed with rabbit anti-GFP (Torrey Pines), rat anti-β-spectrin, or mouse anti-band 3 (BRAC65 and BRIC155 respectively; Bristol Institute for Transfusion Sciences).

## 3 Results

### 3.1 Exported PHISTb proteins localise to the periphery of the host erythocyte

To characterise the localisation of exported PHISTb proteins, we expressed a range PHISTb family members [31] with C-terminal GFP tags, in *P. falciparum* parasites. The cloned genes encoded full-length PF3D7_0201600, PF3D7_0401800 (PFD80), PF3D7_0424600, PF3D7_0532400, PF3D7_0936600, PF3D7_1102500, PF3D7_1252700 and PF3D7_1476200 (see Table 1 for details on alternative gene identifiers).

**Table 1:** Gene identifiers and names of genes used in this study.

Gene ID	Genomic Sequence ID	UniProt ID	Gene Name	Previous ID(s)
*P. falciparum* PHISTb				
PF3D7_0401800	Pf3D7_04_v3	Q8I207	PfD80	MAL4P1.16, PFD0080c
PF3D7_0424600	Pf3D7_04_v3	Q8IFM0	Null	MAL4P1.229, PFD1170c
PF3D7_0532400	Pf3D7_05_v3	Q8I3F0	Null	MAL5P1.315, PFE1605w
PF3D7_1102500	Pf3D7_11_v3	Q8IIX5	GEXP02	PF11_0037
PF3D7_1476200	Pf3D7_14_v3	Q8IK76	Null	PF14_0730, PF14_0731
PF3D7_1252700	Pf3D7_12_v3	Q8I4Q9	Null	2277.t00505, MAL12P1.502, PFL2535w
PF3D7_0201600	Pf3D7_02_v3	O96121	Null	PF02_0016, PFB0080c
PF3D7_0102200	Pf3D7_01_v3	Q8I0U6	RESA	PFA0110w MAL1P1.13

*P. falciparum* PHISTa				
PF3D7_1001300	Pf3D7_10_v3	Q8IK23	Null	PF10_0017
PF3D7_0832200.1	Pf3D7_08_v3	Null	Null	MAL7P1.225

*P. falciparum* PHISTc				
PF3D7_0731100	Pf3D7_07_v3	Q8IBF1	GEXP11	MAL7P1.172
PF3D7_1016600	Pf3D7_10_v3	C6S3C7	Null	PF10_0161, PF10_0161a
PF3D7_0936600^a^	Pf3D7_09_v3	Q8I2F4	GEXP05	PFI1770w

*P. vivax* PHISTb				
PVX_003555	Pv_Sal1_chr04	A5KBH3	Null	Pv003555

*P. knowlesi* PHISTb				
PKH_103230	Pk_strainH_chr10	B3L6R3	Null	PK00_0910c

a The protein encoded by PF3D7_0936600 is classified as a PHISTc protein by Frech et al. [35] but as a PHISTb protein by Sargeant et al. [31].

**Fig. 1:**
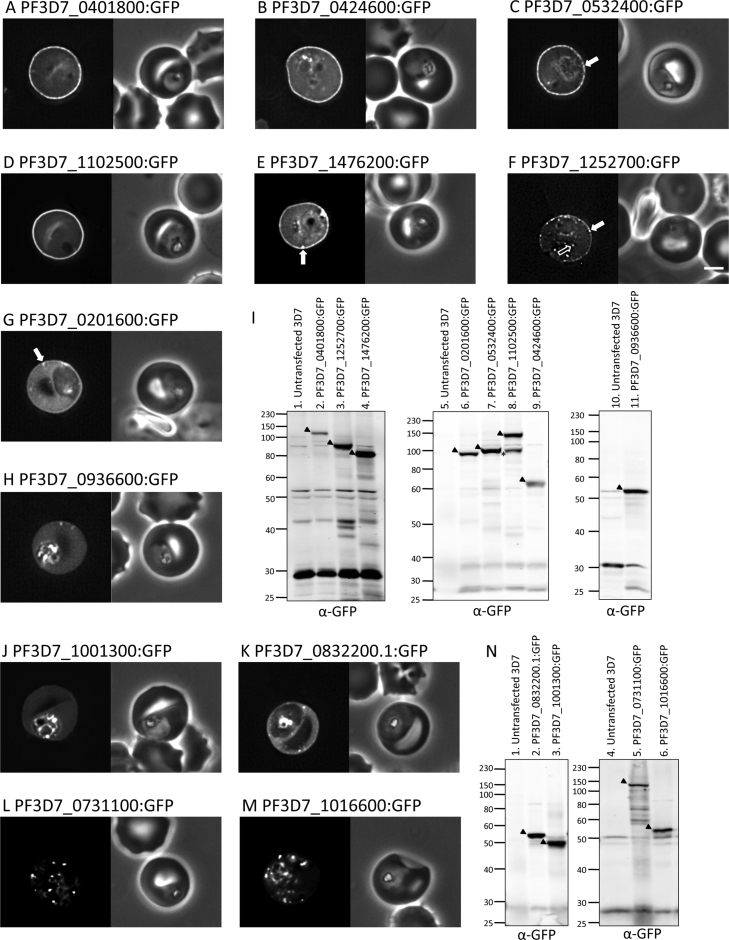
(A–H) Localisation of PHISTb:GFP proteins. The left- and right-hand images show GFP localisation and a phase contrast image, respectively. White filled arrows: peripheral GFP puncta; White unfilled arrow: GFP puncta in host erythocyte cytosol. Scale bar, 2 μm. The identity of each parasite line is indicated above the respective images. (I) Anti-GFP Western blots of schizonts expressing PHISTb:GFP proteins. 2 × 10^6^ schizonts were loaded per lane. The identity of each PHISTb:GFP protein is indicated above each lane. Black arrow: expressed PHISTb:GFP protein. Asterisk: PF3D7_1102500:GFP degradation product. Lanes 1, 5 and 10 contain untransfected parasites. (J–K) Localisation of PHISTa:GFP proteins. Image panels are as in (A–H). PF3D7_1001300:GFP (J); PF3D7_0832200.1:GFP (K). (L–M) Localisation of PHISTc:GFP proteins. Image panels are as in (A–H). PF3D7_0731100:GFP (L); PF3D7_1016600:GFP (M). (N) Anti-GFP Western blots of schizonts expressing PHISTa:GFP and PHISTc:GFP proteins. 2 × 10^6^ schizonts were loaded per lane. The identity of each PHIST:GFP protein is indicated above each lane. Black arrow: expressed protein. Lanes 1 and 4 contain untransfected parasites. For additional images of parasites see also supplementary Figs. 1 and 3.

When expressed in *Plasmodium* parasites, GFP-tagged PF3D7_0401800 (PFD80), PF3D7_0424600, PF3D7_0532400, PF3D7_1102500 and PF3D7_1476200 were all exported to the host cell and displayed a striking localisation at the edge of the host erythrocyte (Fig. 1A–E, respectively; see also [46] and [50] for localisation of PF3D7_0532400). The GFP fluorescence was predominantly localised uniformly at erythrocyte periphery, with some fluorescence apparent in the erythrocyte cytoplasm, and little or no accumulation in the parasite. Quantitation of fluorescence images of parasites expressing PF3D7_0401800 (PFD80), PF3D7_0424600, PF3D7_0532400, PF3D7_1102500 and PF3D7_1476200 showed a 4.47 (S.D. ± 1.43), 2.57 (S.D. ± 0.61), 4.20 (S.D. ± 1.38), 3.75 (S.D. ± 1.32) and 2.24 (S.D. ± 0.47) fold increase in fluorescence intensity at the erythrocyte membrane relative to the erythrocyte cytosol, respectively (supplementary Fig. 1). In addition to the uniformly distributed fluorescence, the PF3D7_0532400:GFP and PF3D7_1476200:GFP parasite lines also exhibited small numbers of bright spots of GFP at the erythrocyte periphery (Fig. 1C and E, respectively, white filled arrows).

PF3D7_1252700:GFP was localised to the erythrocyte periphery (Fig. 1F) and also exhibited peripheral GFP punctae (Fig. 1F, white filled arrow), which were much more numerous than observed for PF3D7_0532400 and PF3D7_1476200, as well as some GFP punctae in the host erythrocyte cytosol (Fig. 1F, white unfilled arrow). Nonetheless, the GFP fluorescence observed for PF3D7_1252700:GFP was clearly peripheral in the host erythrocyte.

PF3D7_0201600:GFP showed some weak accumulation at the erythrocyte periphery, but the majority of the protein was localised in the RBC cytosol (Fig. 1G; supplementary Fig. 1). Consistent with this, the ratio of fluorescence at the erythrocyte periphery relative to the erythrocyte cytoplasm was low (1.14 fold S.D. ± 0.21; supplementary Fig. 1). This protein also exhibited occasional spots of GFP fluorescence in the host cell (Fig. 1G, white filled arrow).

The only PHISTb protein tested that did not exhibit some peripheral localisation in the host erythrocyte was GFP-tagged PF3D7_0936600. This protein was originally classified as a PHISTb protein [31] but has subsequently been reclassified as a PHISTc protein [35]. We find that the GFP tagged protein was localised in the erythrocyte cytosol, not at the host cell periphery (Fig. 1H); PF3D7_0936600:GFP exhibited 0.91 (S.D. ± 0.09) fold fluorescence intensity at the erythrocyte membrane relative to the erythrocyte cytosol (supplementary Fig. 1).

Expression of full-length protein was confirmed for each PHISTb:GFP-expressing parasite line by anti-GFP Western blots of purified schizonts (Fig. 1I). Western blots of each parasite line gave bands of the expected molecular weight; PF3D7_0401800:GFP (PFD80) and PF3D7_1102500:GFP both ran approximately 30 kDa larger than predicted, which is likely due to the presence of repetitive or highly charged sequences. A degradation product of PF3D7_1102500:GFP was also observed (Fig. 1I, lane 8, asterisk).

PF3D7_0401800:GFP (PFD80) was also expressed in *P. falciparum* Dd2 parasites, which have a deletion in chromosome 2 and lack the gene encoding KAHRP. In Dd2 parasites, the protein exhibited robust localisation at the erythrocyte periphery (supplementary Fig. 2) indicating that targeting was not dependent on KAHRP or the presence of assembled knob structures [51].

All of the GFP-tagged PHISTb proteins tested exhibited some degree of peripheral localisation at the edge of the host erythrocyte (with the exception of PF3D7_0936600, which has been reclassified as a PHISTc protein [35]). PHISTb proteins are a sub-class of a larger family of exported proteins that also includes PHISTa and PHISTc proteins. Members of the PHISTa and PHISTc protein sub-classes were also GFP tagged and expressed in *Plasmodium*, in order to assess their localisation.

GFP-tagged PHISTa proteins, PF3D7_1001300 and PF3D7_0832200.1, were efficiently exported into the host erythrocyte and exhibited diffuse fluorescence localisation in the erythrocyte cytosol (Fig. 1J and K, respectively). These proteins exhibited 0.96 (S.D. ± 0.07) and 0.98 (S.D. ± 0.13) fold fluorescence intensity at the erythrocyte membrane relative to the erythrocyte cytosol, respectively (supplementary Fig. 1). Anti-GFP Western blotting of purified schizonts expressing PF3D7_0832200.1:GFP and PF3D7_1001300:GFP confirmed expression of GFP proteins of the correct predicted sizes (Fig. 1N, lanes 2 and 3, respectively).

GFP-tagged PHISTc proteins, PF3D7_0731100 and PF3D7_1016600, were efficiently exported to the host cell where they exhibited bright puncta of GFP fluorescence (Fig. 1L and M, respectively and supplementary Fig. 3). The punctate localisation of PF3D7_0731100:GFP is in keeping with the immunolocalisation of the endogenous protein [18], [38]. The expression of full-length PF3D7_0731100:GFP and PF3D7_1016600:GFP was confirmed by anti-GFP Western blotting of purified schizonts (Fig. 1N, lanes 5 and 6, respectively).

Together these data present a previously undescribed localisation for members of the PHISTb subclass, which is distinct from the members of the PHISTa and PHISTc subclasses tested here.

### 3.2 Solubility profiles of PHISTb proteins localised to the erythocyte periphery support interactions of PHISTb proteins with the erythrocyte cytoskeleton

We next sought to assess the mechanism by which the peripheral PHISTb proteins were targeted to the edge of the host erythrocyte. None of the mature (i.e. post-HT cleaved) PHISTb proteins tested contain strongly predicted transmembrane domains, indicating that they are either peripherally associated with the erythrocyte plasma membrane or associated with the spectrin cytoskeleton. To distinguish between these possibilities, purified schizonts expressing the various constructs were subjected to sequential solubilisation in hypotonic and triton-based buffers. Hypotonic lysis releases soluble, non-membrane-associated material; membrane-associated material remains in the ‘ghost’ pellet. Subsequent solubilisation of ghosts in triton X-100 releases transmembrane and peripherally associated membrane proteins, while the spectrin cytoskeleton and many of its associated proteins remain in the triton-insoluble pellet fraction. Thus, solubilisation of ghosts in triton X-100 allows the assessment of whether proteins are membrane- or cytoskeleton-associated [29].

**Fig. 2:**
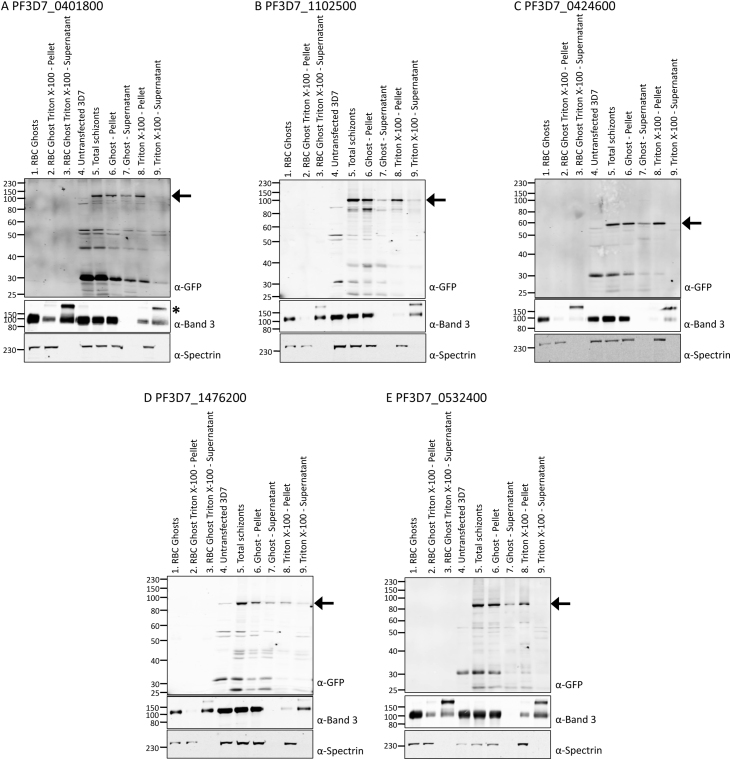
(A–E) Western blots of material from sequential solubilisations of purified schizonts expressing PHISTb:GFP proteins. Material equivalent to 2 × 10^6^ cells was loaded per lane. Upper panel, α-GFP; middle panel, α-Band 3; bottom panel, α-Spectrin. Black arrow indicates expressed PHISTb:GFP protein. Asterisk: Band 3 dimer. Lane 1: uninfected erythrocyte ghost pellet, lane 2: insoluble pellet of 1% triton X-100-solubilised uninfected erythrocyte ghosts, lane 3: supernatant of 1% triton X-100-solubilised uninfected erythrocyte ghosts, lane 4: untransfected schizonts, lane 5: total schizonts expressing PHISTb:GFP protein, lane 6: ghost pellet of hypotonically lysed schizonts expressing PHISTb:GFP protein, lane 7: supernatant of hypotonically lysed schizonts expressing PHISTb:GFP protein, lane 8: insoluble pellet of 1% triton X-100-solubilised ghost pellet of schizonts expressing PHISTb:GFP protein, lane 9: supernatant of 1% triton X-100-solubilised ghost pellet of schizonts expressing PHISTb:GFP protein. The identity of each parasite line is indicated above the respective blots.

Five peripherally localised PHISTb:GFP proteins were tested (PF3D7_0401800:GFP (PFD80), Fig. 2a; PF3D7_1102500, Fig. 2b; PF3D7_0424600, Fig. 2c; PF3D7_1476200, Fig. 2d; PF3D7_0532400, Fig. 2e). Percoll-purified schizonts were first lysed in hypotonic buffer to release soluble proteins. In each case, hypotonic lysis released some of the PHISTb:GFP protein into the ghost supernatant (Fig. 2, α-GFP, lane 7). However, the majority of the protein remained in the ghost pellet (Fig. 2, α-GFP, lane 6). The erythrocyte transmembrane protein Band 3 also remained in the ghost pellet (Fig. 2, α-Band 3, lane 6), as did the cytoskeleton protein, spectrin (Fig. 2, α-spectrin, lane 6).

The ghost pellets were subsequently resuspended in a 1% triton X-100 buffer in order to solubilise transmembrane and membrane-associated proteins. The ghost-associated PHISTb:GFP protein fraction was not released upon solubilisation of the ghost pellet in 1% triton X-100; the proteins remained in the triton X-100 insoluble pellet (Fig. 2, α-GFP, lane 8) with spectrin (Fig. 2, α-spectrin, lane 8). Triton X-100 solubilisation of the ghost pellet did release the majority of the membrane-associated Band 3 into the soluble supernatant (Fig. 2, α-Band 3, lane 9). Upon solubilisation of schizont-ghosts in 1% triton X-100, band 3 appeared as a doublet by Western blotting (Fig. 2A, α-Band 3, asterisk, lane 9). This was also observed for triton X-100-solubilised uninfected erythrocyte ghosts (Fig. 2, α-Band 3, lane 3) and is likely due to incomplete denaturation of a Band 3 dimer in the presence of 1% triton X-100, as seen previously [52].

For each PHISTb:GFP protein tested, a fraction was released upon hypotonic lysis. The remaining protein in the ghost pellet fraction remained insoluble in detergent, indicative of association with components of the triton X-100-insoluble spectrin cytoskeleton.

### 3.3 Localisation of PHISTb proteins at the erythrocyte periphery is dependent on both the PRESAN domain and its preceding sequence

**Fig. 3:**
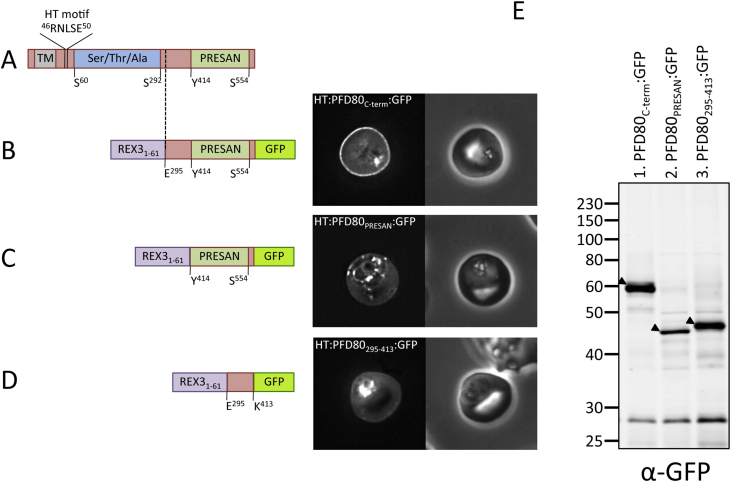
(A) Domain architecture of PF3D7_0401800 (PFD80), not to scale. TM, transmembrane domain/recessed signal sequence; Ser/Thr/Ala refers to serine, threonine and alanine rich-region; PRESAN, domain boundaries defined according to Pfam ID: PF09687. (B–D) Domain architecture and localisation of HT:PFD80:GFP reporter constructs. REX3_1–61_, N-terminal sequence of REX3, including its HT motif, which is sufficient to drive protein export. Residue numbers, below each diagram, refer to position within PFD80. The left- and right-hand images show GFP localisation and a phase contrast image, respectively. The identity of each parasite line is indicated above the respective images. (E) Anti-GFP Western blot of schizonts expressing PFD80:GFP reporter proteins. 2 × 10^6^ schizonts were loaded per lane. The identity of each PFD80:GFP reporter protein is indicated above each lane. Black arrow: expressed protein.

The PHISTb family member PF3D7_0401800 (PFD80) was used initially as a model protein for elucidating the protein region responsible for localisation to the erythrocyte periphery. PFD80 consists of an N-terminal Ser/Ala/Thr-rich region (approximately residues 60–292), followed by a C-terminal PRESAN domain between residues 414 and 554 (ending six residues short of the C-terminus; Fig. 3A). To ascertain the region of PFD80 responsible for peripheral erythrocyte localisation, we generated parasite lines expressing N-terminally truncated fragments of PFD80, tagged C-terminally with GFP. Since the endogenous N-terminus of PFD80 (which contains the HT motif) was removed in these expression constructs, the proteins were targeted for export by appending residues 1–61 of REX3 (PF3D7_0936300/PFI1755c) to their N-termini. Residues 1–61 of REX3 contain a validated HT motif, drive efficient export to the host erythrocyte, and do not contribute to peripheral localisation [12], [31]. Hence, using the validated N-terminus of REX3 to drive reporter export allows assessment of the specific contribution of PFD80 fragments to protein localisation, independently of the N-terminal PFD80 sequences adjacent to the HT motif.

Parasites expressing PFD80 residues 295-end (encompassing the PRESAN domain and 119 preceding residues) fused to GFP exhibited GFP fluorescence at the erythrocyte periphery (HT:PFD80_C-term_:GFP; Fig. 3B). Peripheral fluorescence was 1.97 (S.D. ± 0.49) fold higher than the erythrocyte cytosol (supplementary Fig. 1). Expression of protein was confirmed by Western blotting of percoll-purified schizonts (Fig. 3E, lane 1). This indicates that a region sufficient to localise PFD80 to the erythrocyte periphery exists within the C-terminal portion of the protein, which includes the PRESAN domain.

We postulated that the PRESAN domain alone may be responsible for peripheral localisation. However, parasites expressing the PFD80 PRESAN domain (PFD80 residues 414-end) fused to GFP exhibited diffuse fluorescence throughout the erythrocyte cytoplasm, with some retention inside the parasite (HT:PFD80_PRESAN_:GFP; Fig. 3C). Peripheral fluorescence was 0.96 (S.D. ± 0.10) fold that of the erythrocyte cytosol (supplementary Fig. 1). Thus, in comparison to HT:PFD80_C-term_:GFP (Fig. 3B), the PFD80 PRESAN domain alone was not sufficient to mediate efficient protein targeting to the erythrocyte periphery. Western blotting of purified schizonts confirmed the expression of the HT:PFD80_PRESAN_:GFP protein (Fig. 3E, lane 2).

These data indicate that the sequence preceding the PRESAN domain (residues 295–413), or this sequence together with the PRESAN domain, constitute the functional domain required for peripheral erythrocyte localisation. To test this, parasites were generated which expressed PFD80 residues 295–413 (the region preceding the PRESAN domain), tagged C-terminally with GFP (HT:PFD80_295–413_:GFP). This protein also exhibited diffuse localisation within the erythrocyte cytosol, as well as some retention in the parasite (Fig. 3D and supplementary Fig. 1). Fluorescence at the erythrocyte membrane was 1.01 (S.D. ± 0.13) fold that of the erythrocyte cytosol (supplementary Fig. 1). In comparison to HT:PFD80_C-term_:GFP or full-length PFD80, residues 295–413 are not sufficient to confer efficient peripheral erythrocyte localisation and alone do not fully constitute the functional targeting domain of PFD80. Expression of the protein was confirmed by Western blotting (Fig. 3E, lane 3).

These data show that in the PHISTb protein PFD80, the PRESAN domain and the 119 residues N-terminal to it, together combine to form a functional domain that is sufficient to confer localisation to the periphery of the host erythrocyte. Neither domain alone is functional.

Alignment of all *P. falciparum* PHIST PRESAN domains and the preceding 150 residues (or to the cleaved HT motif, whichever was shorter) and six residues C-terminal to the annotated PRESAN domain, showed that there are regions of sequence homology in the amino acid sequence preceding the PRESAN domain; this is particularly clear in the ∼30 residues immediately preceding the PRESAN domain (supplementary Fig. 4). The sequence conservation in this region and its necessity in addition to the annotated PRESAN domain for peripheral localisation of PHISTb proteins indicate that the N-terminal extension is a functional component of the domain responsible for peripheral targeting. Henceforth we will refer to this combined, functional domain as the ‘extended PRESAN’ domain.

### 3.4 The extended PRESAN domain of RESA independently localises to the periphery of the host erythrocyte

**Fig. 4:**
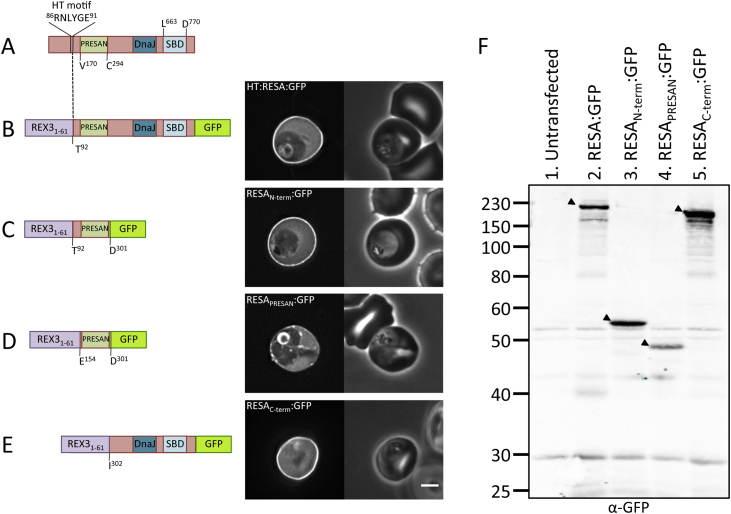
(A) Domain architecture of PF3D7_0102200 (RESA), not to scale. PRESAN, domain boundaries defined according to Pfam ID: PF09687; DnaJ domain as defined by Pfam ID: PF14308; SBD, spectrin-binding domain. (B–E) Domain architecture and localisation of HT:RESA:GFP reporter constructs. REX3_1–61_, N-terminal sequence of REX3, including its HT motif, which is sufficient to drive protein export. Residue numbers, below each diagram, refer to position within full-length RESA. The left- and right-hand images show GFP localisation and a phase contrast image, respectively. Scale bar, 2 μm. The identity of each parasite line is indicated above the respective images. (F) Anti-GFP western blot of schizonts expressing RESA:GFP reporter proteins. 2 × 10^6^ schizonts were loaded per lane. The identity of each RESA:GFP reporter protein is indicated above each lane. Black arrow: expressed protein. Lane 1 contains untransfected parasites.

Ring-infected Erythrocyte Surface Antigen (RESA; PF3D7_0102200) has previously been shown to localise the erythrocyte periphery [40], [41]. The protein contains an N-terminal PRESAN domain (residues 170–294), and a DnaJ domain towards the C-terminus (Fig. 4A). Residues 663–770 bind to spectrin *in vitro* but it has not been demonstrated that this region is sufficient to confer cytoskeletal localisation *in vivo*.

RESA has the atypical HT motif ^86^RNLYGE^91^, which does not support protein export when protein expression is directed by late promoters [53]. To characterise the cytoskeleton targeting elements in RESA, we generated a chimeric RESA reporter construct that could be expressed from a strong, late promoter. This allows a higher protein expression level and hence more brightly fluorescent parasites, and also allows us to assess the localisation of RESA fragments independently of the HT-motif proximal residues of RESA. A ‘full-length’ RESA reporter expressed from a non-RESA promoter was generated by replacing the region of RESA containing the information necessary for export (residues 1–91) with the equivalent sequence from the exported protein REX3 (residues 1–61). This region of REX3 contains a HT motif that supports export when expression is driven by the PfCAM promoter [12]. Thus, this RESA reporter consisted of a validated, exportable N-terminus followed by the portion of RESA from immediately following the RESA HT motif (Thr92) to the end of the protein, with a C-terminal GFP tag (RESA:GFP; Fig. 4B). As expected, RESA:GFP was exported and localised at the erythrocyte periphery (Fig. 4B). Peripheral fluorescence was 2.08 (S.D. ± 0.34) fold higher than the erythrocyte cytosol (supplementary Fig. 1). Western blotting of purified schizonts confirmed expression of the full-length protein (Fig. 4F, lane 2).

Given the observation for PFD80 that the PRESAN domain plus an N-terminal 119 residues were required for efficient localisation at the erythrocyte periphery, we sought to determine whether the extended PRESAN domain of RESA also localised to the erythrocyte periphery. Parasites were generated which expressed RESA residues Thr92-Asp301 (i.e. from immediately following the RESA HT motif to seven residues after the predicted PRESAN domain) with a C-terminal GFP tag (RESA_N-term_:GFP). These parasites exhibited strong GFP fluorescence at the erythrocyte periphery (Fig. 4C). Peripheral fluorescence was 2.35 (S.D. ± 0.43) fold higher than the erythrocyte cytosol (supplementary Fig. 1). Western blotting of purified schizonts confirmed expression of the full-length protein (Fig. 4F, lane 3). The extended PRESAN domain of RESA can confer peripheral localisation in the host erythrocyte independently of the RESA's C-terminal spectrin binding domain. A GFP tagged construct containing the RESA PRESAN domain with only 16 preceding residues (Glu154-Asp301) was expressed in parasites. By comparison to the RESA_N-term_:GFP protein construct (Fig. 4C), the PRESAN domain alone is not efficiently recruited to the erythrocyte periphery (Fig. 4D and supplementary Fig. 1) (ratio of GFP at the membrane relative to the cytoplasm is 1.10 S.D. ± 0.18) indicating that this domain alone does not fully reconstitute the targeting activity of full-length RESA or RESA_N-term_:GFP. Expression of the protein was confirmed by Western blotting of purified schizonts (Fig. 4F, lane 4). Together, these data are consistent with our observations for PFD80 and indicate that the PRESAN domain of RESA plus additional N-terminal sequence can confer peripheral localisation in the erythrocyte.

The C-terminal fragment of RESA (Ile302-end) was also cloned and expressed in parasites with a C-terminal GFP tag (RESA_C-term_:GFP). This region includes the segment that binds to the erythrocyte cytoskeleton protein spectrin *in vitro* [43]. In keeping with the *in vitro* data, this construct also localised to the erythrocyte periphery (Fig. 4E), with a peripheral fluorescence intensity 3.08 (S.D. ± 0.78) fold higher than the erythrocyte cytosol (supplementary Fig. 1). Expression of the full-length protein was confirmed by Western blotting of purified schizonts (Fig. 4F, lane 5)

Together, our experiments using both PFD80 and RESA show that the PHISTb extended PRESAN domain, but not the PRESAN domain alone, is sufficient to mediate localisation to the host erythrocyte periphery.

In the case of RESA, this extended region functions independently and in addition to the previously identified spectrin-binding region located towards the C-terminus of the protein. The ability of the RESA C-terminal spectrin-binding region to independently localise to the erythrocyte periphery has been confirmed using a GFP reporter here in live parasites. Together, these data show that RESA contains multiple independent regions with the capacity to localise to the erythrocyte periphery.

### 3.5 *P. vivax* and *P. knowlesi* relatives of PHISTb proteins also localise to the erythrocyte periphery

**Fig. 5:**
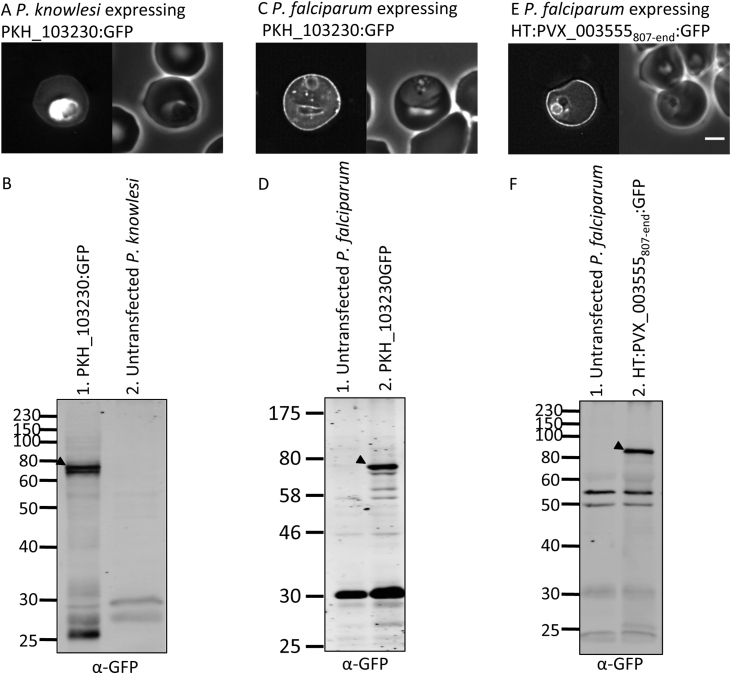
(A) Localisation of PKH_103230:GFP expressed in *P. knowlesi.* The left- and right-hand images show GFP localisation and a phase contrast image, respectively. Scale bar, 2 μm. (B) Western blot of *P. knowlesi* schizonts expressing PKH_103230:GFP (Lane 1). 2 × 10^6^ schizonts were loaded per lane. Black arrow: PKH_103230:GFP. Lane 2 contains untransfected parasites. (C) Localisation of PKH_103230:GFP expressed in *P. falciparum*. Image panels are as in (A). (D) Western blot of *P. falciparum* schizonts expressing PKH_103230 (lane 2). Lane 1 contains untransfected parasites. Black arrow: PKH_103230:GFP. (E) Localisation of HT:PVX_033555_807-end_:GFP. Image panels are as in (A). (F) Anti-GFP western blot of schizonts expressing HT:PVX_033555_807-end_:GFP (Lane 2). 2 × 10^6^ schizonts were loaded per lane. Black arrow: expressed protein. Lane 1 contains untransfected parasites.

To establish whether peripherally localised PHIST proteins were present in other human-infective *Plasmodium* species, we tested the localisation of PHISTb homologues from *P. knowlesi and P. vivax* [35]. Transfection and culturing of *P. knowlesi* in human blood has recently been established [47]. Therefore we were able to test the localisation of a *P. knowlesi* PHISTb protein, PKH_103230 [35], in human erythrocytes infected with either transfected *P. knowlesi* or *P. falciparum* parasites. When expressed by *P. knowlesi* grown in human erythrocytes, GFP-tagged PKH_103230 (PKH_103230:GFP) exhibited localisation at the host erythrocyte periphery, as well as some retention inside the parasite (Fig. 5A). Peripheral fluorescence intensity was 2.20 (S.D. ± 0.71) fold higher than the erythrocyte cytoplasm (supplementary Fig. 1). Expression of the protein was confirmed by Western blotting (Fig. 5B, lane 1). Likewise, when PKH_103230:GFP was expressed in *P. falciparum* parasites, the protein also localised to the erythrocyte periphery (Fig. 5C), with a fluorescence intensity 1.93 (S.D. ± 0.48) fold higher than that of the erythrocyte cytosol (supplementary Fig. 1). Western blotting confirmed the expression of full-length protein (Fig. 5D, lane 2).

The *P. vivax* PHISTb protein, PVX_003555 is 1122 residues in length. We expressed a GFP-tagged fragment of the protein extending from residue 807 to the end of the protein; this encompassed the PRESAN domain and the preceding 150 residues only (i.e. an extended PRESAN domain). As this fragment of the protein lacks its normal N-terminal export signal, the protein was fused to the N-terminal 61 residues of REX3 (HT:PVX_003555_807-end_:GFP). HT:PVX_003555_807-end_:GFP was robustly exported and localised to the periphery of the host erythrocyte (Fig. 5E). GFP fluorescence intensity at the erythrocyte periphery was 2.69 (S.D. ± 1.07) fold greater than that of the erythrocyte cytosol (supplementary Fig. 1). The expression of a protein of the expected size was confirmed by Western blotting of purified schizonts (Fig. 5F, lane 2).

These data indicate that the targeting function of the extended PRESAN domain in PHISTb proteins is a conserved feature used by multiple human malaria parasite species.

## 4 Discussion

PHIST proteins constitute a major family of exported proteins that is expanded in the human-infectious malaria species *P. falciparum*, *P. vivax* and *P. knowlesi* [31]. Almost 20% of predicted HT motif-containing exported proteins encoded by the *P. falciparum* genome contains a PRESAN domain [31]. Understanding the function of this domain is key to our understanding of host cell modification and pathogenesis of malaria.

Studies of several PHISTa, b, and c family proteins indicate that they play important but diverse roles in the host cell [16], [25], [39]. However, little is known about the role of the PRESAN domains that are the defining feature of each family. Protein-protein interactions have been described for a small number of PHIST family members; the PRESAN domain of PHISTa protein PF3D7_0402000, which is exported and localised at the parasitophorous vacuole membrane, interacts with Band 4.1 in a two-hybrid assay, and the PHISTc protein PF3D7_0936800 interacts with the C-terminal domain of PfEMP1 *in vitro* [25], [39]. Microarray analyses indicate that essential and non-essential PHISTb proteins are expressed in the blood stages [54] and also in gametocytes [55].

Here, we identify novel PHISTb proteins that localise to the erythrocyte periphery. Furthermore, truncations of a PF3D7_0401800 (PFD80) reporter protein allowed us to define a novel region sufficient to confer peripheral localisation in the host erythrocyte. This region of PFD80, comprising both the PRESAN domain and an additional N-terminal flanking sequence, is sufficient to target to the erythrocyte periphery; neither domain alone is functional. The boundaries of the PRESAN domain have been defined based on sequence analysis [32]. We identified additional weak sequence conservation extending N-terminal to the PRESAN domain. Our data show that this region N-terminal to the PRESAN domain contributes, along with the PRESAN domain, to a functional peripheral-targeting domain in PHISTb proteins. The precise role of the region N-terminal to the PRESAN domain in peripheral localisation requires further investigation. There is also some weak sequence conservation preceding the PRESAN domains in other PHIST families (supplementary Fig. 4). As the function of these proteins is not well characterised it remains to be determined whether the regions N-terminal to the PRESAN domains of PHISTa/c proteins also contribute to their function.

The detergent insolubility of the PHISTb proteins that we characterised suggests that they are cytoskeleton-associated. Detergent insolubility is a characteristic of known cytoskeleton associated proteins such as RESA and MESA [27], [42]. Consistent with our results, several of the PHISTb proteins that we find to be detergent insoluble, have also been found in the proteomes of detergent resistant membranes [56]. Interestingly, in parasites isolated from placentae, the PF3D7_0936900 (PFI1785w; PHISTb) transcript is specifically upregulated [57], [58] and the protein is found in detergent resistant membranes [59] suggesting that it is cytoskeleton associated and could play a role in cytoadherence during pregnancy-associated malaria.

We also show that the extended PRESAN domain of RESA, a known cytoskeleton-associated protein, efficiently targets to the erythrocyte periphery. This region is distinct from, and functions independently of the previously characterised C-terminal spectrin-binding region [43] that we also show here is functional *in vivo*. Therefore, we have shown for the first time that RESA contains multiple domains that target to the erythrocyte periphery; the extended PRESAN domain and the C-terminal spectrin-binding domain [43]. A recent study of the protein PF3D7_0532400, which contains an N-terminal PRESAN domain and is peripherally localised ([46] and Fig. 1c), suggests that it also contains an additional C-terminal domain that interacts with the cytoskeleton. These observations evoke a model whereby RESA and potentially other PHISTb proteins could link multiple cytoskeletal proteins or their domains, thus altering the rigidity of infected erythrocytes [46], [60]. In the case of RESA, our data suggest the protein may enhance the mechanical stability of the cytoskeleton not only by using the C-terminal spectrin-binding domain to stabilise the tetrameric form of spectrin [43] but also by further cross-linking elements of the cytoskeleton. Whilst this paper was under review, Oberli et al. [50], showed that the PHISTb domain of PF3D7_0532400 interacts with the cytoplasmic tail of PfEMP1. Although the functions of RESA and PF3D7_0532400 are different it appears that in both cases they may function by mediating interactions between multiple cytoskeleton- or cytoskeleton-associated proteins.

Merozoite expressed surface antigen (MESA) contains a short 19 amino acid MEC domain that interacts with the erythrocyte cytoskeleton protein band 4.1 [29]. Several PHISTb proteins, not including the PHISTb proteins characterised in this study, contain sequences C-terminal to the PRESAN domain, that have similarity to the MEC domain [30]. Although the localisation of these proteins has not been tested *in vivo*, it is possible that some of these proteins could crosslink elements of the erythrocyte cytoskeleton by forming a protein bridge between band 4.1 and the binding partner of the extended PRESAN domain in PHISTb proteins.

Cytoskeleton-associated proteins, such as KAHRP, MESA, and RESA, have been extensively characterised, but none is essential for parasite survival. PFD80, which we show here to be targeted to the erythrocyte periphery, is known to be essential for survival of blood stage parasites [18]. Given the essential nature of PFD80 *in vitro*, these data suggest that cytoskeleton modifications may be important for parasite survival; several other PHISTb proteins are also essential but it is not known whether they are similarly targeted [16]. It is unclear why PFD80 is essential for parasite survival per se but it is possible that is plays a role in stabilising the integrity of an infected cell or even modifying the properties of the erythrocyte plasma membrane that is immediately proximal to the cytoskeleton.

Several functions have been attributed to non-essential PHISTb proteins although the localisation of these proteins has not been described [16]. Deletion of the gene PF3D7_0220100 (PFB0920w) leads to an increase in the rigidity of infected cells [16] and deletion of PF3D7_1401600 (PF14_0018) or RESA leads to a decrease in cell rigidity [16], [60]. These phenotypes are entirely consistent with the deletion of proteins that might be localised to the erythrocyte cytoskeleton and regulate its function. Furthermore, deletion of PF3D7_0424600 (PFD1170c) leads to loss of cytoskeleton-associated knob structures on the surface of infected erythrocytes and a partial disruption of cytoadherence under flow conditions [16]. Consistent with this, we show here for the first time that PF3D7_0424600 localises to the erythrocyte periphery and has a solubility profile consistent with it interacting with the cytoskeleton.

The interaction of the extended PRESAN domains alone with cytoskeleton or associated protein complexes may be sufficient to alter their properties; this mode of action may be most relevant to small PHISTb proteins, such as PF3D7_0424600, that contain only an extended PRESAN domain and little other protein sequence. In addition, extended PRESAN domains in multi-domain PHISTb proteins may localise other functional domains to the erythrocyte periphery where they can perform their function. For example, the PHISTb protein PF3D7_0220100 is a large protein that contains a PRESAN domain and a J-domain. The latter domain could mediate localised activation of Hsp70 chaperones suggesting that it may remodel protein complexes in the cell periphery.

Finally, we show that the genomes of malaria parasites *P. vivax* and *P. knowlesi* encode PHISTb proteins that are recruited to the erythrocyte periphery. Both parasites modify the properties of their host cell. *P. vivax*-infected erythrocytes, exhibit increased flexibility [61], and caveolae-like membrane invaginations, referred to as Schüffner's dots, are assembled in the erythrocyte membrane [36]. Notably, the PHISTc protein PvPHIST/CVC-8195 is localised to Schüffner's dots in *P. cynomolgi* infected erythrocytes. The rigidity of *P. knowlesi* infected cells is increased [62]. Although the parasites do not make knob structures, occasional electron dense invaginations of the erythrocyte plasma membrane are seen, infected cells become sequestered in the microcirculation of many tissues [63], and are able to cytoadhere to specific protein ligands such as ICAM-1, VCAM and CD36 [64]. Our data suggest that PHISTb proteins could contribute to these phenotypes by interacting with and modifying the cytoskeleton or plasma membrane of infected cells. The *P. knowlesi* genome encodes ∼10 other PHISTb proteins that are closely related to PKH_103230 [35] as well as numerous more distantly related PHIST proteins. Although the *P. vivax* protein PVX_003555 clusters closely with PHISTb proteins from *P. falciparum*, other PHIST proteins from *P. vivax* are more distantly related; further experiments will be required to test whether these proteins are also peripherally targeted.

*P. vivax, P. falciparum*, and *P. knowlesi* share relatively few conserved exported proteins but all contain functional PHISTb proteins within their genomes. This would suggest that the although the proteins may be recruited to the periphery of infected erythrocytes by multiple protein interactions, at least one of these interactions will be with conserved host cytoskeletal elements or one of the few conserved exported parasite proteins. In this context we find that PFD80 is efficiently localised to the erythrocyte membrane in Dd2 parasites that lack KAHRP and assembled knob structures.

In summary, we show that the extended PRESAN domain, found in PHISTb proteins, is sufficient to target exported parasite proteins to the erythrocyte periphery. This domain architecture is found in many multi-domain PHISTb proteins and in multiple parasite species. Outside of the shared extended PRESAN domain other diverse domains are found in these proteins including J-domains, J-like domains, cytoskeleton interacting domains, PRESAN domains from other PHIST families, as well as other uncharacterised domains. The extended PRESAN domain may be used to recruit functionally diverse proteins to the periphery of the infected erythrocyte to modify properties of both the erythrocyte cytoskeleton and plasma membrane thus playing a key role in parasite survival and virulence.

## References

[1] (2013).

[2] Singh B., Daneshvar C. (2013). Human infections and detection of *Plasmodium knowlesi*. Clin Microbiol Rev.

[3] Hiller N.L., Bhattacharjee S., van Ooij C., Liolios K., Harrison T., Lopez-Estrano C. (2004). A host-targeting signal in virulence proteins reveals a secretome in malarial infection. Science.

[4] Marti M., Good R.T., Rug M., Knuepfer E., Cowman A.F. (2004). Targeting malaria virulence and remodeling proteins to the host erythrocyte. Science.

[5] Boddey J.A., Hodder A.N., Gunther S., Gilson P.R., Patsiouras H., Kapp E.A. (2010). An aspartyl protease directs malaria effector proteins to the host cell. Nature.

[6] Knuepfer E., Rug M., Klonis N., Tilley L., Cowman A.F. (2005). Trafficking of the major virulence factor to the surface of transfected *P. falciparum*-infected erythrocytes. Blood.

[7] Przyborski J.M., Miller S.K., Pfahler J.M., Henrich P.P., Rohrbach P., Crabb B.S. (2005). Trafficking of STEVOR to the Maurer's clefts in *Plasmodium falciparum*-infected erythrocytes. EMBO J.

[8] Chang H.H., Falick A.M., Carlton P.M., Sedat J.W., DeRisi J.L., Marletta M.A. (2008). N-terminal processing of proteins exported by malaria parasites. Mol Biochem Parasitol.

[9] Osborne A.R., Speicher K.D., Tamez P.A., Bhattacharjee S., Speicher D.W., Haldar K. (2010). The host targeting motif in exported *Plasmodium* proteins is cleaved in the parasite endoplasmic reticulum. Mol Biochem Parasitol.

[10] Russo I., Babbitt S., Muralidharan V., Butler T., Oksman A., Goldberg D.E. (2010). Plasmepsin V licenses *Plasmodium* proteins for export into the host erythrocyte. Nature.

[11] Grüring C., Heiber A., Kruse F., Flemming S., Franci G., Colombo S.F. (2012). Uncovering common principles in protein export of malaria parasites. Cell Host Microbe.

[12] Tarr S.J., Cryar A., Thalassinos K., Haldar K., Osborne A.R. (2013). The C-terminal portion of the cleaved HT motif is necessary and sufficient to mediate export of proteins from the malaria parasite into its host cell. Mol Microbiol.

[13] Gehde N., Hinrichs C., Montilla I., Charpian S., Lingelbach K., Przyborski J.M. (2009). Protein unfolding is an essential requirement for transport across the parasitophorous vacuolar membrane of *Plasmodium falciparum*. Mol Microbiol.

[14] de Koning-Ward T.F., Gilson P.R., Boddey J.A., Rug M., Smith B.J., Papenfuss A.T. (2009). A newly discovered protein export machine in malaria parasites. Nature.

[15] Heiber A., Kruse F., Pick C., Gruring C., Flemming S., Oberli A. (2013). Identification of new PNEPs indicates a substantial non-PEXEL exportome and underpins common features in *Plasmodium falciparum* protein export. PLoS Pathog.

[16] Maier A.G., Rug M., O’Neill M.T., Brown M., Chakravorty S., Szestak T. (2008). Exported proteins required for virulence and rigidity of *Plasmodium falciparum*-infected human erythrocytes. Cell.

[17] Kraemer S.M., Smith J.D. (2006). A family affair: var genes, PfEMP1 binding, and malaria disease. Curr Opin Microbiol.

[18] Maier A.G., Cooke B.M., Cowman A.F., Tilley L. (2009). Malaria parasite proteins that remodel the host erythrocyte. Nat Rev Microbiol.

[19] Goldberg D.E., Cowman A.F. (2010). Moving in and renovating: exporting proteins from *Plasmodium* into host erythrocytes. Nat Rev Microbiol.

[20] Haldar K., Mohandas N. (2007). Erythrocyte remodeling by malaria parasites. Curr Opin Hematol.

[21] Boddey J.A., Cowman A.F. (2013). *Plasmodium* nesting: remaking the erythrocyte from the inside out. Annu Rev Microbiol.

[22] Glenister F.K., Coppel R.L., Cowman A.F., Mohandas N., Cooke B.M. (2002). Contribution of parasite proteins to altered mechanical properties of malaria-infected red blood cells. Blood.

[23] Crabb B.S., Cooke B.M., Reeder J.C., Waller R.F., Caruana S.R., Davern K.M. (1997). Targeted gene disruption shows that knobs enable malaria-infected red cells to cytoadhere under physiological shear stress. Cell.

[24] Oh S.S., Voigt S., Fisher D., Yi S.J., LeRoy P.J., Derick L.H. (2000). *Plasmodium falciparum* erythrocyte membrane protein 1 is anchored to the actin-spectrin junction and knob-associated histidine-rich protein in the erythrocyte skeleton. Mol Biochem Parasitol.

[25] Mayer C., Slater L., Erat M.C., Konrat R., Vakonakis I. (2012). Structural analysis of the *Plasmodium falciparum* Erythrocyte Membrane Protein 1 (PfEMP1) intracellular domain reveals a conserved interaction epitope. J Biol Chem.

[26] Langreth S.G., Peterson E. (1985). Pathogenicity, stability, and immunogenicity of a knobless clone of *Plasmodium falciparum* in Colombian owl monkeys. Infect Immunol.

[27] Lustigman S., Anders R.F., Brown G.V., Coppel R.L. (1990). The mature-parasite-infected erythrocyte surface antigen (MESA) of *Plasmodium falciparum* associates with the erythrocyte membrane skeletal protein, band 4.1. Mol Biochem Parasitol.

[28] Bennett B.J., Mohandas N., Coppel R.L. (1997). Defining the minimal domain of the *Plasmodium falciparum* protein MESA involved in the interaction with the red cell membrane skeletal protein 4.1. J Biol Chem.

[29] Black C.G., Proellocks N.I., Kats L.M., Cooke B.M., Mohandas N., Coppel R.L. (2008). In vivo studies support the role of trafficking and cytoskeletal-binding motifs in the interaction of MESA with the membrane skeleton of *Plasmodium falciparum*-infected red blood cells. Mol Biochem Parasitol.

[30] Kilili G.K., LaCount D.J. (2011). An erythrocyte cytoskeleton-binding motif in exported *Plasmodium falciparum* proteins. Eukaryot Cell.

[31] Sargeant T., Marti M., Caler E., Carlton J., Simpson K., Speed T. (2006). Lineage-specific expansion of proteins exported to erythrocytes in malaria parasites. Genome Biol.

[32] Oakley M.S.M., Kumar S., Anantharaman V., Zheng H., Mahajan B., Haynes J.D. (2007). Molecular factors and biochemical pathways induced by febrile temperature in intraerythrocytic *Plasmodium falciparum* parasites. Infect Immun.

[33] Pain A., Bohme U., Berry A.E., Mungall K., Finn R.D., Jackson A.P. (2008). The genome of the simian and human malaria parasite *Plasmodium knowlesi*. Nature.

[34] Carlton J.M., Adams J.H., Silva J.C., Bidwell S.L., Lorenzi H., Caler E. (2008). Comparative genomics of the neglected human malaria parasite *Plasmodium vivax*. Nature.

[35] Frech C., Chen N. (2013). Variant surface antigens of malaria parasites: functional and evolutionary insights from comparative gene family classification and analysis. BMC Genomics.

[36] Akinyi S., Hanssen E., Meyer E.V., Jiang J., Korir C.C., Singh B. (2012). A 95 kDa protein of *Plasmodium vivax* and *P. cynomolgi* visualized by three-dimensional tomography in the caveola–vesicle complexes (Schuffner's dots) of infected erythrocytes is a member of the PHIST family. Mol Microbiol.

[37] Mantel P.-Y., Hoang A.N., Goldowitz I., Potashnikova D., Hamza B., Vorobjev I. (2013). Malaria-infected erythrocyte-derived microvesicles mediate cellular communication within the parasite population and with the host immune system. Cell Host Microbe.

[38] Regev-Rudzki N., Wilson D.W., Carvalho T.G., Sisquella X., Coleman B.M., Rug M. (2013). Cell–cell communication between malaria-infected red blood cells via exosome-like vesicles. Cell.

[39] Parish L.A., Mai D.W., Jones M.L., Kitson E.L., Rayner J.C. (2013). A member of the *Plasmodium falciparum* PHIST family binds to the erythrocyte cytoskeleton component band 4.1. Malar J.

[40] Brown G.V., Culvenor J.G., Crewther P.E., Bianco A.E., Coppel R.L., Saint R.B. (1985). Localization of the ring-infected erythrocyte surface antigen (RESA) of *Plasmodium falciparum* in merozoites and ring-infected erythrocytes. J Exp Med.

[41] de Azevedo M.F., Gilson P.R., Gabriel H.B., Simoñes R.F., Angrisano F., Baum J. (2012). Systematic analysis of FKBP Inducible degradation domain tagging strategies for the human malaria parasite *Plasmodium falciparum*. PLoS ONE.

[42] Foley M., Tilley L., Sawyer W.H., Anders R.F. (1991). The ring-infected erythrocyte surface antigen of *Plasmodium falciparum* associates with spectrin in the erythrocyte membrane. Mol Biochem Parasitol.

[43] Pei X., Guo X., Coppel R., Bhattacharjee S., Haldar K., Gratzer W. (2007). The ring-infected erythrocyte surface antigen (RESA) of *Plasmodium falciparum* stabilizes spectrin tetramers and suppresses further invasion. Blood.

[44] Da Silva E., Foley M., Dluzewski A.R., Murray L.J., Anders R.F., Tilley L. (1994). The *Plasmodium falciparum* protein RESA interacts with the erythrocyte cytoskeleton and modifies erythrocyte thermal stability. Mol Biochem Parasitol.

[45] Silva M.D., Cooke B.M., Guillotte M., Buckingham D.W., Sauzet J.P., Le Scanf C. (2005). A role for the *Plasmodium falciparum* RESA protein in resistance against heat shock demonstrated using gene disruption. Mol Microbiol.

[46] Proellocks N.I., Herrmann S., Buckingham D.W., Hanssen E., Hodges E.K., Elsworth B. (2014). A lysine-rich membrane-associated PHISTb protein involved in alteration of the cytoadhesive properties of *Plasmodium falciparum*-infected red blood cells. FASEB J.

[47] Moon R.W., Hall J., Rangkuti F., Ho Y.S., Almond N., Mitchell G.H. (2013). Adaptation of the genetically tractable malaria pathogen *Plasmodium knowlesi* to continuous culture in human erythrocytes. Proc Natl Acad Sci.

[48] Nkrumah L.J., Muhle R.A., Moura P.A., Ghosh P., Hatfull G.F., Jacobs W.R. (2006). Efficient site-specific integration in *Plasmodium falciparum* chromosomes mediated by mycobacteriophage Bxb1 integrase. Nat Methods.

[49] Deitsch K., Driskill C., Wellems T. (2001). Transformation of malaria parasites by the spontaneous uptake and expression of DNA from human erythrocytes. Nucleic Acids Res.

[50] Oberli A., Slater L.M., Cutts E., Brand F., Mundwiler-Pachlatko E., Rusch S. (2014). A *Plasmodium falciparum* PHIST protein binds the virulence factor PfEMP1 and comigrates to knobs on the host cell surface. FASEB J.

[51] Rug M., Prescott S.W., Fernandez K.M., Cooke B.M., Cowman A.F. (2006). The role of KAHRP domains in knob formation and cytoadherence of *P. falciparum*-infected human erythrocytes. Blood.

[52] Toye A.M., Ghosh S., Young M.T., Jones G.K., Sessions R.B., Ramaugé M. (2005). Protein-4.2 association with band 3 (AE1, SLCA4) in *Xenopus* oocytes: effects of three natural protein-4.2 mutations associated with hemolytic anemia. Blood.

[53] Rug M., Wickham M.E., Foley M., Cowman A.F., Tilley L. (2004). Correct promoter control is needed for trafficking of the ring-infected erythrocyte surface antigen to the host cytosol in transfected malaria parasites. Infect Immun.

[54] Bozdech Z., Llinás M., Pulliam B.L., Wong E.D., Zhu J., DeRisi J.L. (2003). The transcriptome of the intraerythrocytic developmental cycle of *P. falciparum*. PLoS Biol.

[55] Silvestrini F., Bozdech Z., Lanfrancotti A., Di Giulio E., Bultrini E., Picci L. (2005). Genome-wide identification of genes upregulated at the onset of gametocytogenesis in *Plasmodium falciparum*. Mol Biochem Parasitol.

[56] Sanders P.R., Cantin G.T., Greenbaum D.C., Gilson P.R., Nebl T., Moritz R.L. (2007). Identification of protein complexes in detergent-resistant membranes of *Plasmodium falciparum* schizonts. Mol Biochem Parasitol.

[57] Tuikue Ndam N., Bischoff E., Proux C., Lavstsen T., Salanti A., Guitard J. (2008). *Plasmodium falciparum* transcriptome analysis reveals pregnancy malaria associated gene expression. PLoS ONE.

[58] Francis S.E., Malkov V.A., Oleinikov A.V., Rossnagle E., Wendler J.P., Mutabingwa T.K. (2007). Six genes are preferentially transcribed by the circulating and sequestered forms of *Plasmodium falciparum* parasites that infect pregnant women. Infect Immun.

[59] Fried M., Hixson K.K., Anderson L., Ogata Y., Mutabingwa T.K., Duffy P.E. (2007). The distinct proteome of placental malaria parasites. Mol Biochem Parasitol.

[60] Mills J.P., Diez-Silva M., Quinn D.J., Dao M., Lang M.J., Tan K.S. (2007). Effect of plasmodial RESA protein on deformability of human red blood cells harboring *Plasmodium falciparum*. Proc Natl Acad Sci U S A.

[61] Suwanarusk R., Cooke B.M., Dondorp A.M., Silamut K., Sattabongkot J., White N.J. (2004). The deformability of red blood cells parasitized by *Plasmodium falciparum* and *P. vivax*. J Infect Dis.

[62] Miller L.H., Usami S., Chien S. (1971). Alteration in the rheologic properties of *Plasmodium knowlesi*—infected red cells. A possible mechanism for capillary obstruction. J Clin Invest.

[63] Miller L.H., Fremount H.N., Luse S.A. (1971). Deep vascular schizogony of *Plasmodium knowlesi* in *Macaca mulatta*. Distribution in organs and ultrastructure of parasitized red cells. Am J Trop Med Hyg.

[64] Fatih F.A., Siner A., Ahmed A., Woon L.C., Craig A.G., Singh B. (2012). Cytoadherence and virulence – the case of *Plasmodium knowlesi* malaria. Malar J.

