# Supplementary material for: A conserved domain targets exported PHISTb family proteins to the periphery of Plasmodium infected erythrocytes
Source: Mol Biochem Parasitol. 2014 Aug;196(1):29–40. doi: 10.1016/j.molbiopara.2014.07.011 (PMC4165601; doi:10.1016/j.molbiopara.2014.07.011)
Supplement: Supplementary file 1 [file mmc1.pdf]

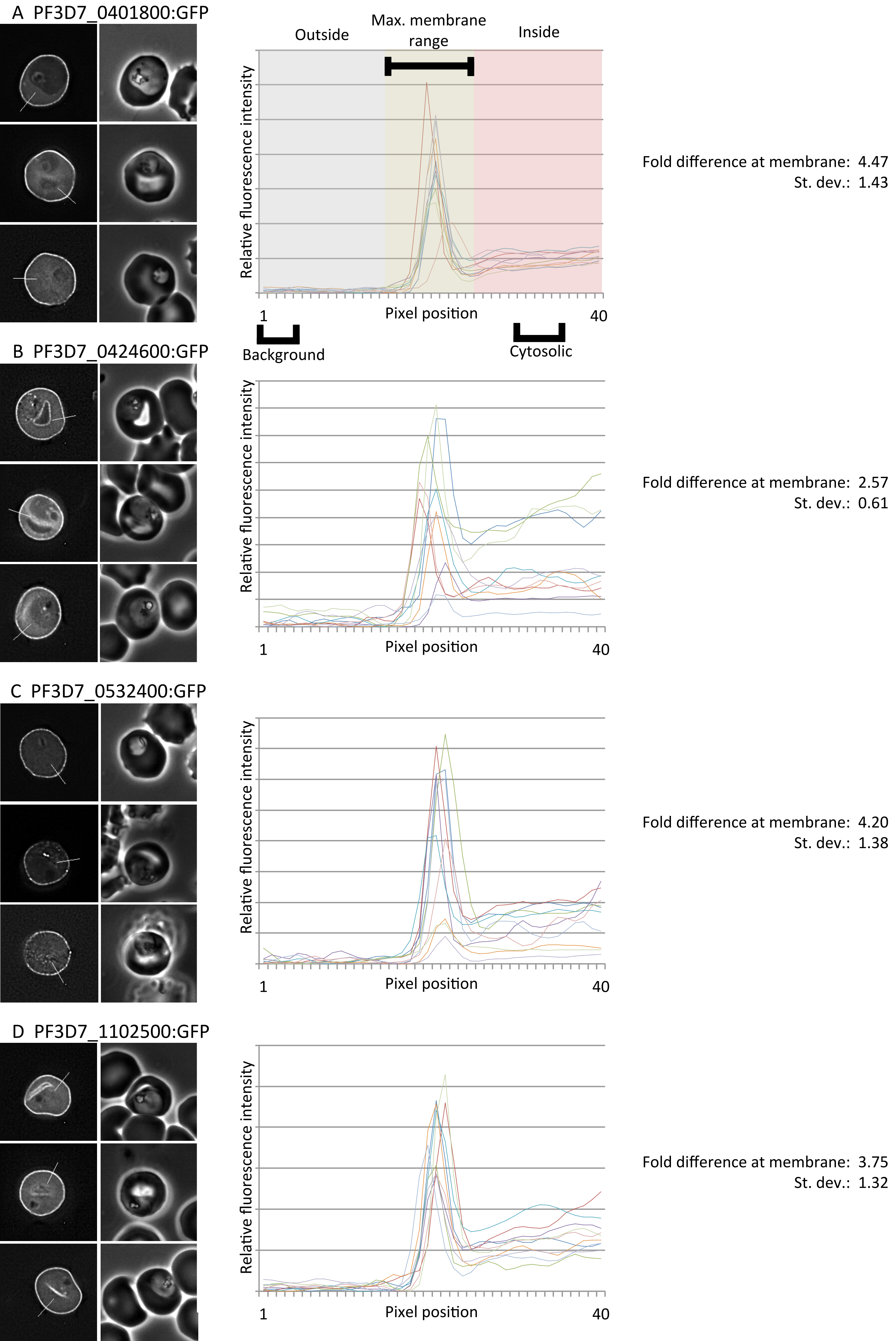
A PF3D7_0401800:GFP
Outside
Max. membrane range
Inside
Relative fluorescence intensity
1
Pixel position
40
Background
Cytosolic
Fold difference at membrane: 4.47
St. dev.: 1.43
B PF3D7_0424600:GFP
Relative fluorescence intensity
1
Pixel position
40
Fold difference at membrane: 2.57
St. dev.: 0.61
C PF3D7_0532400:GFP
Relative fluorescence intensity
1
Pixel position
40
Fold difference at membrane: 4.20
St. dev.: 1.38
D PF3D7_1102500:GFP
Relative fluorescence intensity
1
Pixel position
40
Fold difference at membrane: 3.75
St. dev.: 1.32

E PF3D7_1476200:GFP

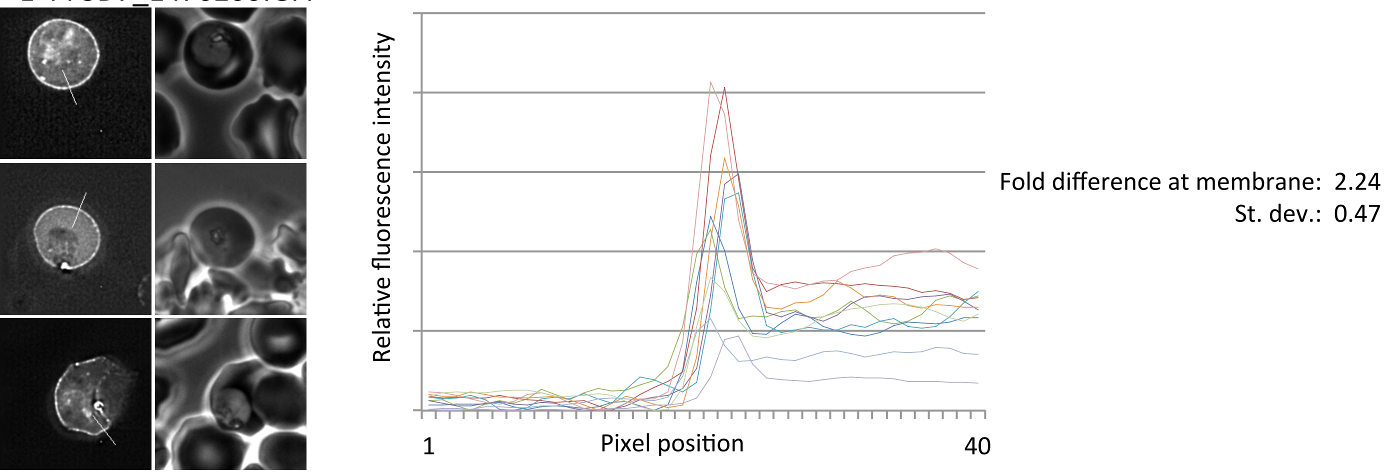

F PF3D7_0201600:GFP

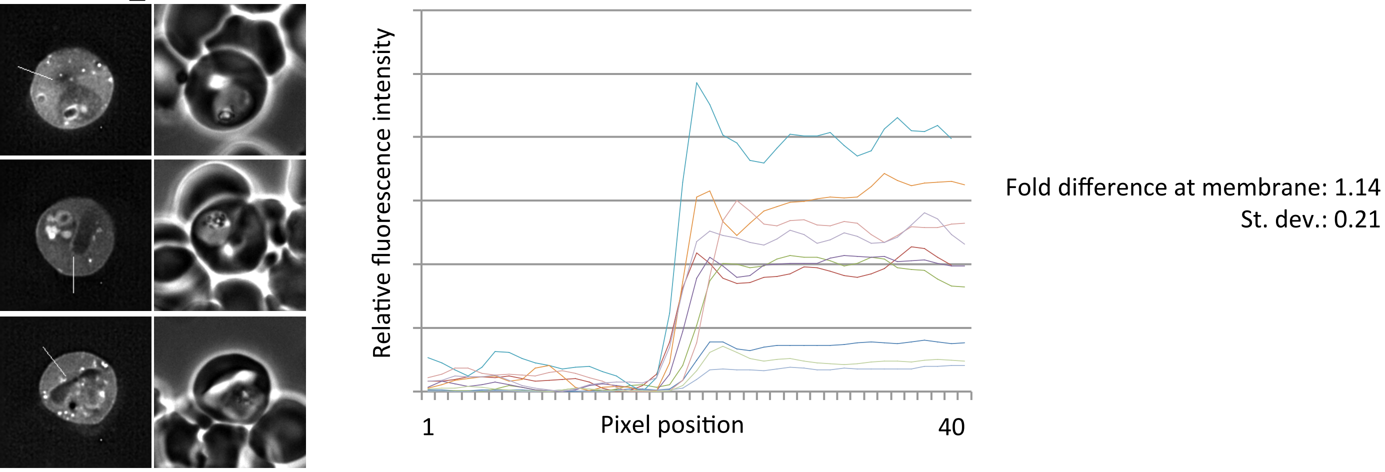

G PF3D7_0936600:GFP

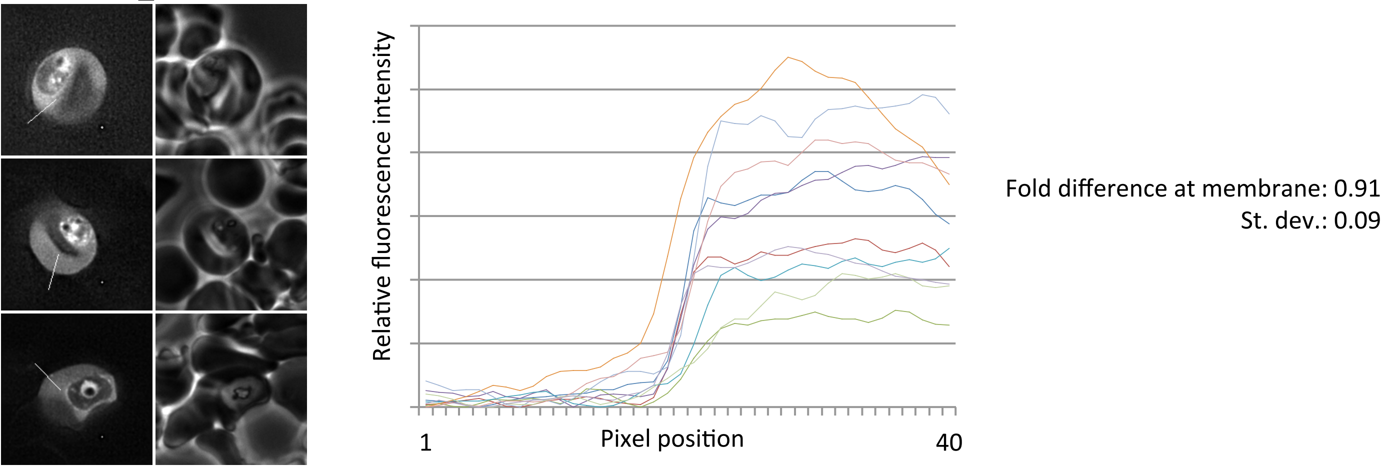

H PF3D7_1001300:GFP

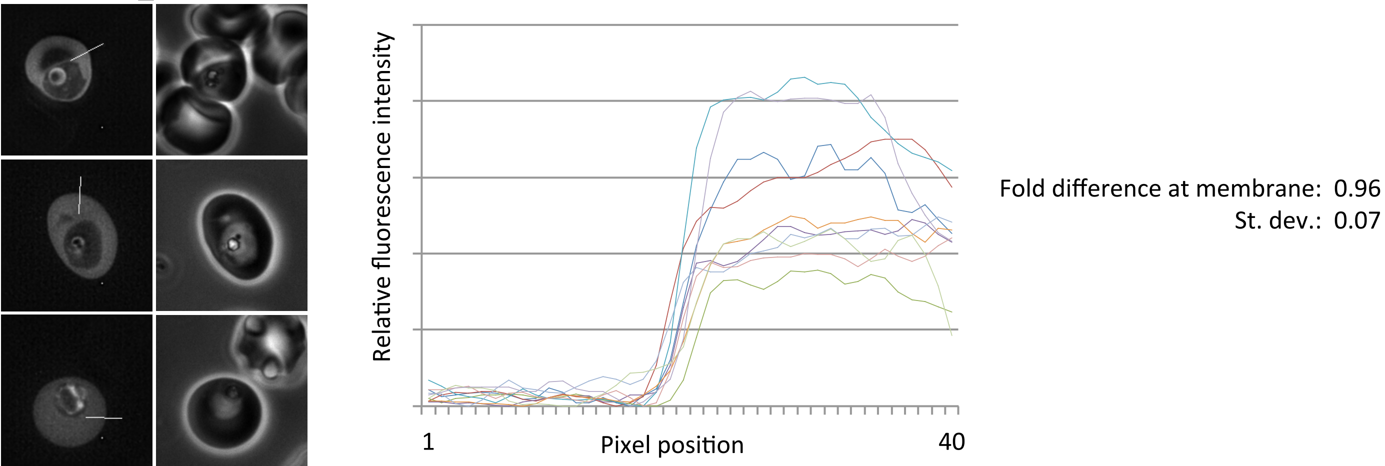

I PF3D7_0832200.1:GFP

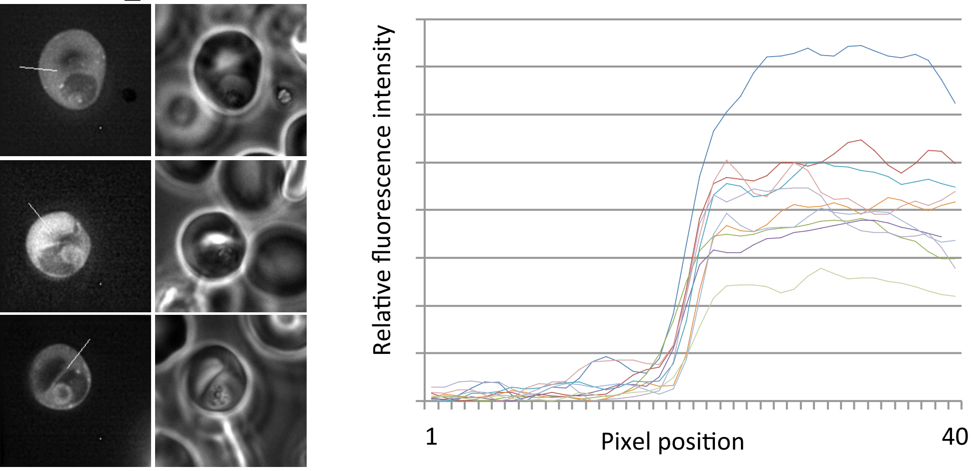

Fold difference at membrane: 0.98
St. dev.: 0.13

J HT:PFD80$_{C\text{-}term}$:GFP

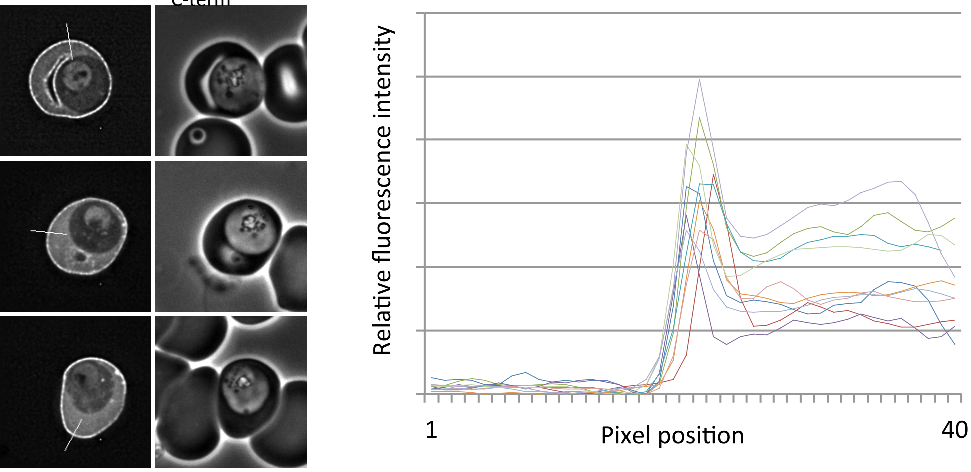

Fold difference at membrane: 1.97
St. dev.: 0.49

K HT:PFD80$_{PRESAN}$:GFP

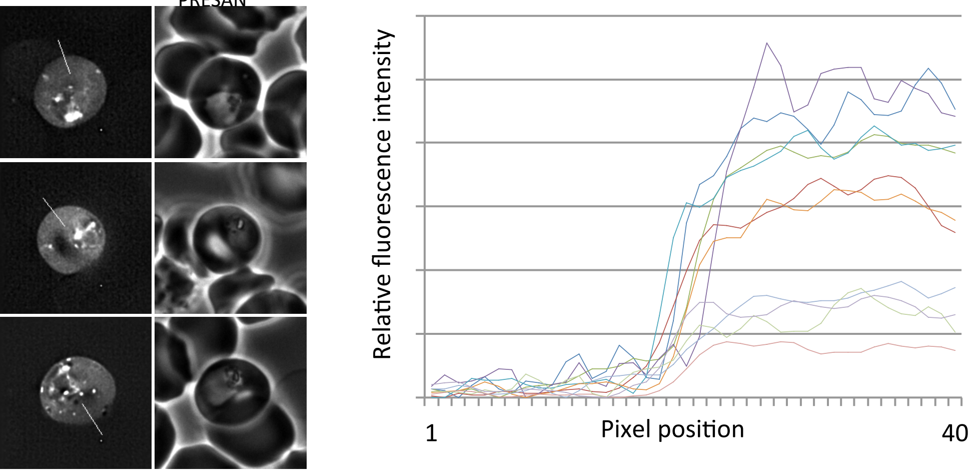

Fold difference at membrane: 0.96
St. dev.: 0.10

L HT:PFD80$_{295\text{-}413}$:GFP

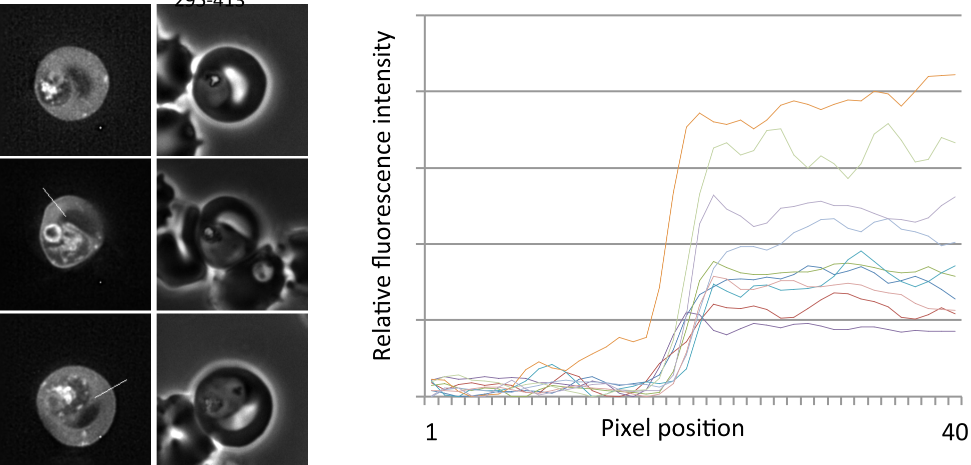

Fold difference at membrane: 1.01
St. dev.: 0.13

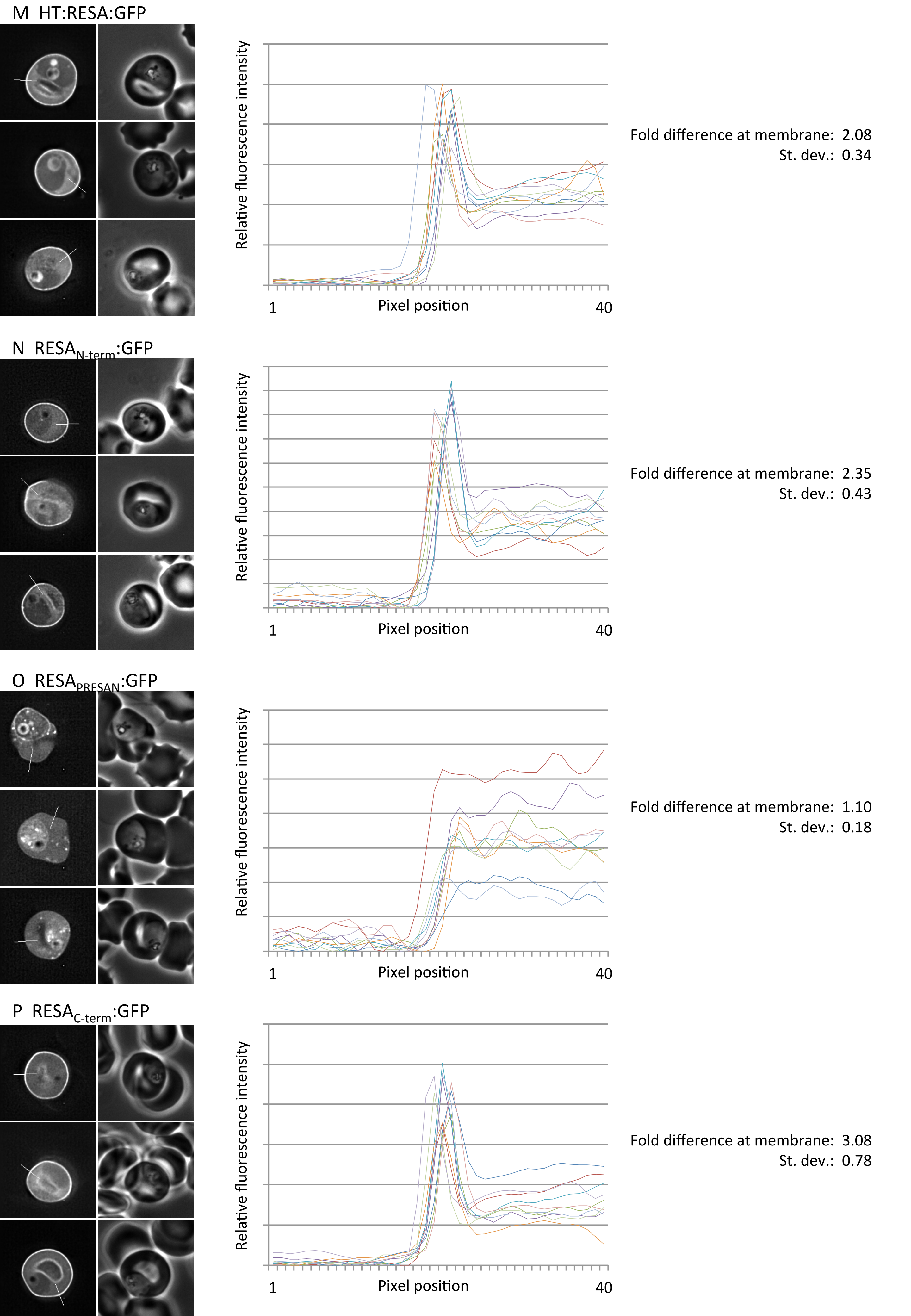
M HT:RESA:GFP
Relative fluorescence intensity
Pixel position
1
40
Fold difference at membrane: 2.08
St. dev.: 0.34
N RESA$_{N\text{-term}}$:GFP
Relative fluorescence intensity
Pixel position
1
40
Fold difference at membrane: 2.35
St. dev.: 0.43
O RESA$_{PRESAN}$:GFP
Relative fluorescence intensity
Pixel position
1
40
Fold difference at membrane: 1.10
St. dev.: 0.18
P RESA$_{C\text{-term}}$:GFP
Relative fluorescence intensity
Pixel position
1
40
Fold difference at membrane: 3.08
St. dev.: 0.78

Q PKH_103230:GFP expressed in *P. knowlesi*

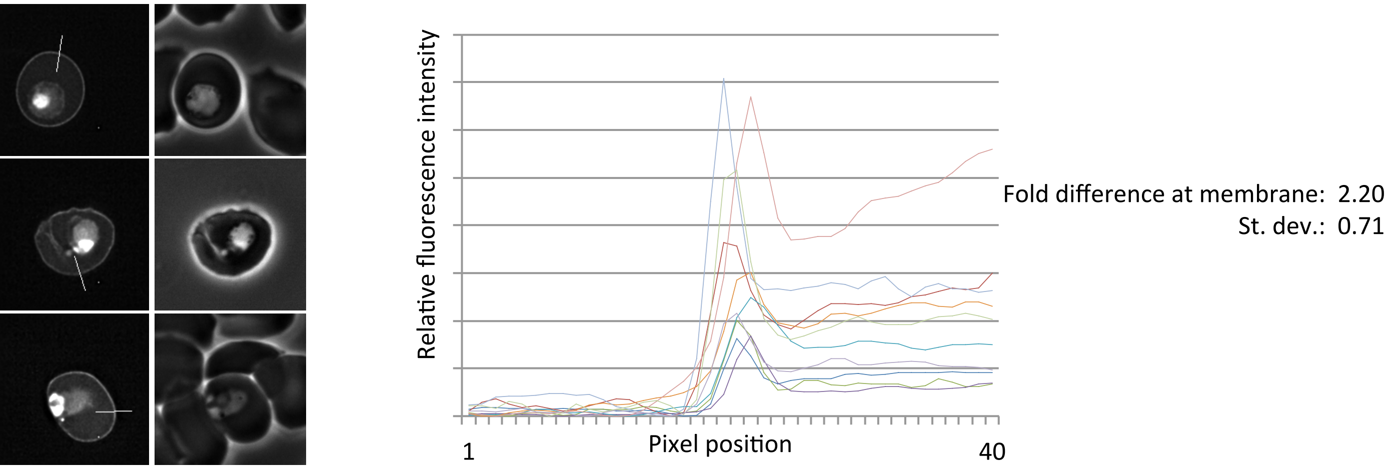

R PKH_103230:GFP expressed in *P. falciparum*

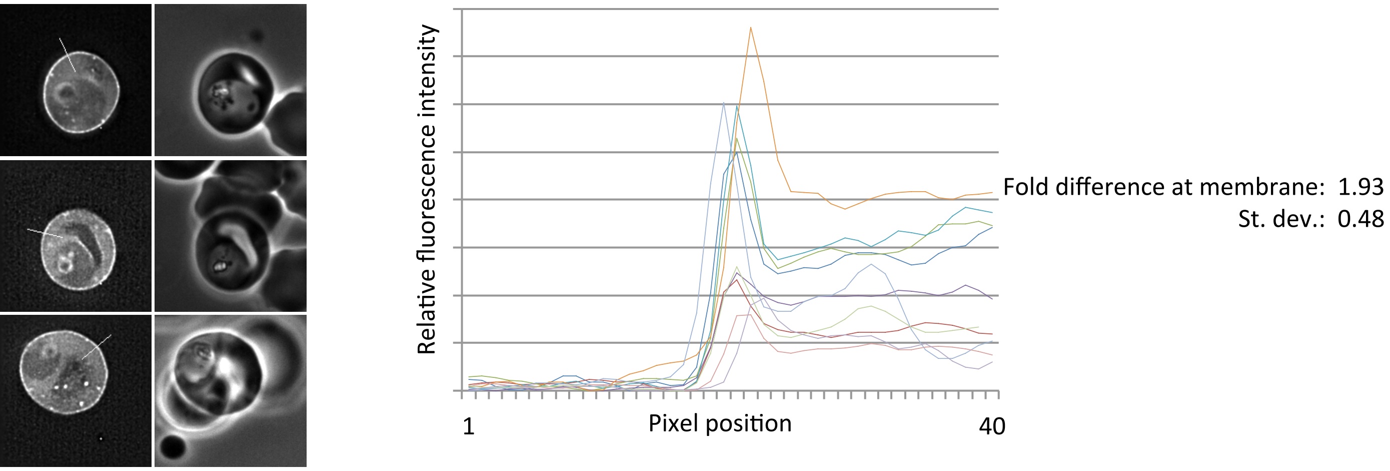

S HT:PVX_003555$_{807\text{-end}}$:GFP

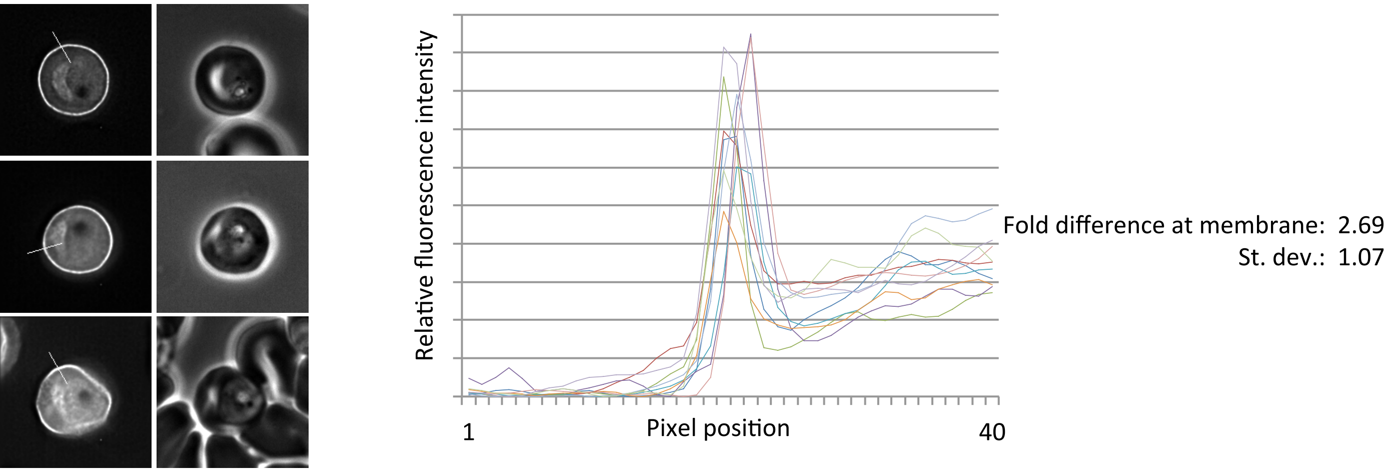

Supplementary figure 1

Additional images of parasites expressing PHIST:GFP proteins. The left- and right-hand images show GFP localisation and a phase contrast image, respectively. The white line on each image is a 40 pixel line used for extraction of relative fluorescence intensity profiles. Plots show the relative fluorescence intensity profiles of 10 cells for each respective cell line (the lowest pixel intensity value was set as zero for the purpose of plotting data). The position of the pixels along the line relative to the infected erythrocyte are highlighted in (A). The region used for calculating mean background fluorescence (pixels 1-5), the range used for indentifying the maximum intensity pixel at the membrane (between pixels 15 and 25) and the region used for calculating mean cytosolic flourescence (pixels 30-35) are marked in (A). The mean fold difference and standard deviation in fluorescence intensity are also shown for each cell line. The identity of each parasite line is indicated above the respective images.
