# Supplementary material for: A conserved domain targets exported PHISTb family proteins to the periphery of Plasmodium infected erythrocytes
Source: Mol Biochem Parasitol. 2014 Aug;196(1):29–40. doi: 10.1016/j.molbiopara.2014.07.011 (PMC4165601; doi:10.1016/j.molbiopara.2014.07.011)
Supplement: Supplementary file 2 [file mmc2.pdf]

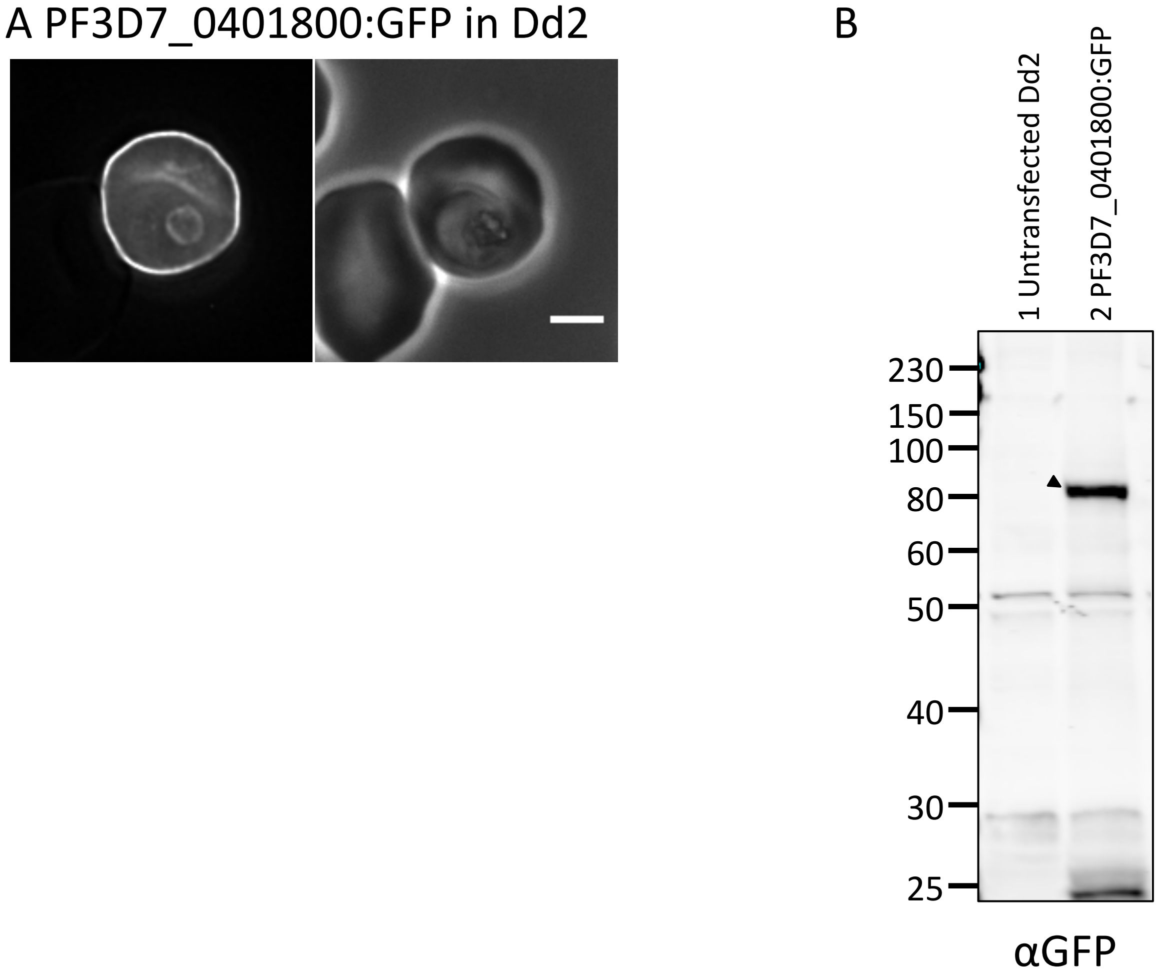

Supplementary figure 2
(A) PF3D7_0401800:GFP (PFD80) expressed in *P. falciparum* Dd2. The left- and right-hand images show GFP localisation and a phase contrast image, respectively. Scale bar, 2 µm.
(B) Anti-GFP Western blot of *P. falciparum* Dd2 schizonts expressing PF3D7_0401800:GFP (PFD80)(lane 2). 2 x 10^6 schizonts were loaded per lane. Black arrow: expressed protein. Lane 1 contains untransfected *P. falciparum* Dd2 parasites.
