# Supplementary material for: A conserved domain targets exported PHISTb family proteins to the periphery of Plasmodium infected erythrocytes
Source: Mol Biochem Parasitol. 2014 Aug;196(1):29–40. doi: 10.1016/j.molbiopara.2014.07.011 (PMC4165601; doi:10.1016/j.molbiopara.2014.07.011)
Supplement: Supplementary file 3 [file mmc3.pdf]

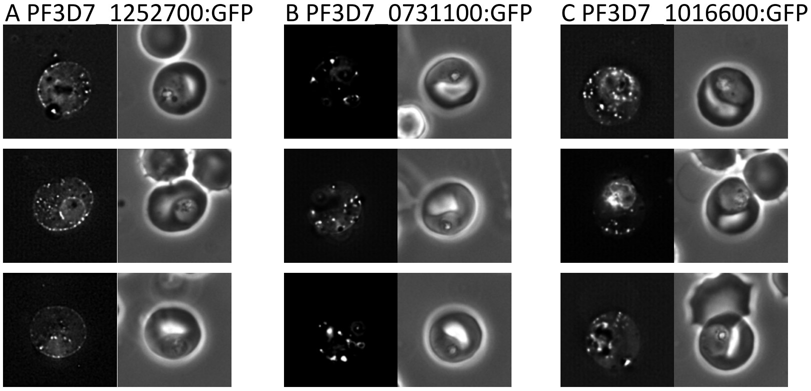

Supplementary figure 3

Additional images of localisation of PHIST:GFP reporter constructs depicted in figure 1. The left- and right-hand images show GFP localisation and a phase contrast image, respectively. The identity of each parasite line is indicated above the respective images.
