# Supplementary material for: A conserved domain targets exported PHISTb family proteins to the periphery of Plasmodium infected erythrocytes
Source: Mol Biochem Parasitol. 2014 Aug;196(1):29–40. doi: 10.1016/j.molbiopara.2014.07.011 (PMC4165601; doi:10.1016/j.molbiopara.2014.07.011)
Supplement: Supplementary file 4 [file mmc4.pdf]

```
  PF3D7_1253800 -------------------------------------------------------------------------------- 0
  PF3D7_1479200 -------------------------------------------------------------------------------- 0
  PF3D7_0424900 -------------------------------------------------------------------------------- 0
  PF3D7_1001100_1 -------------------------------------------------------------------------------- 0
  PF3D7_0425400 -------------------------------------------------------------------------------- 0
  PF3D7_0832300 -------------------------------------------------------------------------------- 0
  PF3D7_1478000 -------------------------------------------------------------------------------- 0
  PF3D7_0402000 -----------------------------------------------------------------SELQKGNQPCLRHTN 15
  PF3D7_1301500 -------------------------------------------------------------------------------- 0
  PF3D7_0832200_1 -------------------------------------------------------------------------------- 0
  PF3D7_1001300 -------------------------------------------------------------------------------- 0
  PF3D7_0115100 -------------------------------------------------------------------------------Y 1
  PF3D7_0800600 -------------------------------------------------------------------------------Y 1
  PF3D7_1477700 -------------------------------------------------------------------------------- 0
  PF3D7_0102200 -------------------------------------------------------------------------------- 0
  PF3D7_1149200 -------------------------------------------------------------------------------- 0
  PF3D7_0201700 -------------------------------------------------------------------------------- 0
  PF3D7_0220100 -------------------------------------------------------------------------------- 0
  PF3D7_1038800 -------------------------------------------------------------------------------- 0
B PF3D7_0424600 -------------------------------------------------------------------------------- 0
B PF3D7_0601500 ---------------------------------------------LSERKISSSEESLYVKRAPFSCFYLFYLMISRRLP 35
B PF3D7_0631100 ----------------------------------------------SERKISSSEESLYVKRAPFSCFYLFYLMISRRLP 34
B PF3D7_0201600 -------------------------------------------------------------------------------- 0
B PF3D7_1477500 -------------------------------------------------------------------------------- 0
B PF3D7_1252700 -------------------------------------------------------------------------------- 0
B PF3D7_1401600 -------------------------------------------------------------------------------- 0
  PF3D7_0831200 -------------------------------------------------------------------------------- 0
B PF3D7_1201000_1 -------------------------------------------------------------------------------- 0
  PF3D7_1201100 -------------------------------------------------------------------------------- 0
B PF3D7_1372100 ------------KILKDYMNDTVQIDVETIFGDDSFRKDDEDKINISYVKEYKPKDDNVKKILDDILSYKNIKDINKLIN 68
B PF3D7_0402100 -------------------------------------------------------------------------------- 0
  PF3D7_1372000 ------------------------------KHYSGKHNKDTSYDALEEDSNDSEEDSENDSEDESEVESENESENESEDQ 50
  PF3D7_1253100 -------------------------------------------------------------------------CEIKKGG 7
B PF3D7_1102500 ---------KKDEKVALEKSTDNISNIKSKVVGIKEIDEEGMESISKTIDNSKTSEVVEEKKTEDVDSLNECLYFYPNDG 71
B PF3D7_0731300 -------------------------------------------------------------------------------- 0
B PF3D7_1252800 -------------------------------------------------------------------------------- 0
B PF3D7_0424800 --------------------------------STNESADVVVEKPMEEKLNNTNENIDETHEESDPSNSTEETVKKCISN 48
B PF3D7_0831000 -------------------------------------------------------------------------------- 0
B PF3D7_0937000 ----------------------------------------------------------SEREELVEILGEYSDGKSNVLE 22
B PF3D7_0532400 -------------------------------------------------------------------------------- 0
B PF3D7_0532300 -------------------------------------------------------------------------------- 0
B PF3D7_0401800 -------------------TTRLPSTGSTIRFPSTGSSRLEDYEKEGSNVDITEEEEKEAESVDDNNKEGNESDNIYCML 61
B PF3D7_1476300 -------------------------------------------------------------------------------- 0
  PF3D7_0936600 -------------------------------------------------------------------------------- 0
  PF3D7_1201200 -------------------------------------------------------------------------------- 0
  PF3D7_0731100 -----------------------STTSLGLGYNDAQNYKGKNRDDYNYEELNEWDYRNYNNYDGNYEVYQDSATMETNYY 57
B PF3D7_1476200 ---------------EKNGFQFDEDDEEDEEDDGDDDDDDDDDYDDEDEEEEDMKKKNYKNVPTKIDVKKPNNTNKVCDN 65
  PF3D7_0219700 -----------------------------------------------------------------------------SER 3
  PF3D7_0219800 ----------------------------------------AQKFKKLIQKKILGKFFSSRKNEKGVPRENVDSTTTSYNS 40
  PF3D7_1016600 -------------------------------------------------------------------------------- 0
  PF3D7_1200900 ------------------NRDYFRKKNHELNYACNNNNNIYAYKDMHLDNFDDVDNFDYFDNIDDVNNMEGVDNFGDVHK 62
  PF3D7_1001700 -------------------------------------------------------------------------------- 0
  PF3D7_1016500 -----------------CYRCRNEKNHIRKNNEETNIKLKTEHKTHKESSNSSINELSSYKNRKIKNINEDKSFSEEQTK 63
  PF3D7_1016700 ------------------SNSSTQKKKKKRSKVPDSRKHSRTLRKRDDNNDDDDNDDDVNNDDDVNNDDICNDSEYEFPT 62
  PF3D7_0936800 -------------------------------------------------------------------------------- 0
  PF3D7_0801000 ------------------TKGFNPMEQRREEDRGHMGGRGSRYPEEERYNYNNKRSNSIPEGRNYEENAYERGGGNNKWD 62
  PF3D7_0202100 -------------------------------------------------------------------------------- 0
  PF3D7_1016800 -----------------------------------------------------------------------------TEV 3
  PF3D7_0830600 ---------------DEEEDKRLKEAYERILRKRRGISGRHPYHILTSSCNEFNSNNIINEEELDHVELIQNEMDSDSYN 65
  PF3D7_1148700 -------------------------------------------------------------------------------- 0
  PF3D7_0424000 ---------------NLYNNKLKSNPYDSKFRNDIYTSRGSHNTKESKEKSSLRDGVSRNNASDVSRNHLREPLYKRFEK 65
  PF3D7_0532200 -------------------------------------------------------------------------------- 0
  PF3D7_1001800 -------------------------------------------------------------------------------- 0
  PF3D7_1201000_2 --------------------DEKEKDEKKKDEKEKDEKEKDEKEKKVSNIKNNLRAVPQNSGSNFDEFLDVKEANEIVQD 60
  PF3D7_1477400 -------------------------------------------------------------------------------- 0
  PF3D7_1477300 -------------------------------------------------------------------------------S 1
```

```
   PF3D7_1253800   --VQVEKNNKGSKVKESLKLKKEDSDKSIDNVNDIKYNEQKIQENIYTINNDIE--------------------------  52
   PF3D7_1479200   --VQVEKNNKGSKVKESLKLKKEDSDKSIDNVNDIKYNEQKIQENIYTINNDIE--------------------------  52
   PF3D7_0424900   --VQVEKS-KGQKSKKHLKHRK-DVNKTKDNENNIKGNAQQVDENKNSIKNNGE--------------------------  50
   PF3D7_1001100_1 --VQVEKN-KDSKKKRNLKHKKVDMNKTKSNENNNKCNEENVEKNEHSISNDFE--------------------------  51
   PF3D7_0425400   --VQVEKN-KTSKRKKYLKHKKEDIDKTKANANDLKCNELCLEENKHSINNDLG--------------------------  51
   PF3D7_0832300   --CEIENG--NNEKRQNIMNRLDQTNESSKNSNN---NKQITLENK----------------------------------  39
   PF3D7_1478000   --TEVKTP-KNEKFKKSFFKNKKKCDNE-NEVNNKDTNKRNTGEPKSCCSGNSE--------------------------  50
   PF3D7_0402000   RTDNSKMNKVKNNNQTENNDNKKKLGNKEDNQGKNKNNNNKEKQNDINKRG-----------------------------  66
   PF3D7_1301500   ----------------HNNMNKNNMDNADMSKNDMYIHNKHNDSGYISKT------------------------------  34
   PF3D7_0832200_1 --SETESVEHSGLSNTSEDVELENGLEENNNNNISCDLEGSNSEDE----------------------------------  45
   PF3D7_1001300   --SEIEKENYTSIESNNEKSKLN---------------------DE----------------------------------  23
   PF3D7_0115100   DLQRIVKGRKKDSKLKNKSEGTNEQQNNKNNGNIVKNEELLSRSNKKCLKVDLNRE------------------------  57
   PF3D7_0800600   DLQRIVKGRKKDSKLKNKSEGTNEQQNNKNNGNIVKNEELLSRSNKKCLKVDLNRE------------------------  57
   PF3D7_1477700   ----SAKRRRMNYNDDDAYDESHYNHNSTNNGNSDKAEDIWNGIDSIFNRIMQN--------------------------  50
   PF3D7_0102200   ---YGETLPVNPYADSENPIVVSQVFGLPFEKPTFTLESPPDIDHTN-ILGFNE--------------------------  50
   PF3D7_1149200   ---YCETLPINPYADSENPIVVSQVFGLPSEKPTFTLEGTPDIDHTN-ILGFNE--------------------------  50
   PF3D7_0201700   ---SEKDNLSNSLEQNGEPVVIGQFFSLPNGK-SISISDDIFIDEEQSIVNFID--------------------------  50
   PF3D7_0220100   ---CESVRSKNSNDTIKEPVLKNKVFSLPNEK-KLTKSEDICDNNVNCIFKFNE--------------------------  50
   PF3D7_1038800   ----YSTESLNREKTNTEKTIISKVNLLPNEI-ILSNLKNENNKREESVLEFNE--------------------------  49
B  PF3D7_0424600   SEGQSTDEYSESSSEYVENPVIGSTFMLPNEN-RLNTIGNLFEGEKYNILEDTQ--------------------------  53
B  PF3D7_0601500   KIKRINSICKDNNEDIVFLDELRHVSDISDKKEIEKIFEKSYEMNECNIFTMNE--------------------------  89
B  PF3D7_0631100   KIKRINSICKDNNEDIVFLDELRHVSDISDKKEIEKIFEKSYEMNECNIFTMNE--------------------------  88
B  PF3D7_0201600   ---------------SSKEPSLDDFFKFYKKN-SYKLSESFMD-TKNMLTKKMKIEKS-AFNKYS---------------  47
B  PF3D7_1477500   ---SDNEKEKEKEKEKEKEKENKEFYKCYKKKGIKKLTIEYTEYMQKALKRKLKAGKNEAFKKYS---------------  62
B  PF3D7_1252700   ---FETNNGNNGYVSSLTNKILNNMNKGPKKEVEEKNAEKILGFDDKNILEALDLFINNIKN------------------  59
B  PF3D7_1401600   ---YENKGVTYESNVEVHDNFLNKLYMSPNVENNGIKEDNVFGNTDLGGMESHEKFKEKIRR------------------  59
   PF3D7_0831200   ---YATERFNEIFGIPSKDVLRSRSKIERYEEDDVILINRFVDEDEYNAFLYIK--------------------------  51
B  PF3D7_1201000_1 ---SSANNGNEYDILDLGIPSVFSVFSLSKEEMMTLLKDVYGKTKKVNLSDEEK--------------------------  51
   PF3D7_1201100   ----CEAETINISRSNVNTPLTSNFFSLLYNTSPCILDDVFENKEEYMLKYIQE--------------------------  50
B  PF3D7_1372100   YFRKYSGVSNKLIVECVNYLLYHLNTEEANNKSLDVLIEELMQCDENNIFKKNK--------------------------  122
B  PF3D7_0402100   -YHETSSLESDEVEEHGKLYKLTDVINYGEGSLLKQVPKEESENSNNDIFEYYK--------------------------  53
   PF3D7_1372000   SENDTENDTENETEQESEEQSQNESDGESENESEEESDDEPVKKNAPKNKEDTKNEEKKDLRQGKGDIIRKTLNN-----  125
   PF3D7_1253100   FESFLNKICKYKLWNKKDDLNKKKIKKKKNKENNEDSKEQNINKNKDSCNNVTD--------------------------  61
B  PF3D7_1102500   DSMNLENVLKHIMEYDNSENTIDDLSEEENYNQLFNLLYRAFQNKFYNIFKLHE--------------------------  125
B  PF3D7_0731300   ---SEVESLQSSNYSEVMKNVKDGNDSSSEELDNTNVPTSKPKKKLSAVFKENK--------------------------  51
B  PF3D7_1252800   -------CTPDIIDNVVNVKIEEEENINKENDNVIKSIKSIKYPDNVNLYNALR--------------------------  47
B  PF3D7_0424800   ILDMTCDSRGHALILANNILRSQNVNEEVIILNEHDLLSQFLNGDVSNIFSDNK--------------------------  102
B  PF3D7_0831000   ----NGEYSDNPLFDYKLFKYHDILYKGILNKELSEEFDLLFSDNTN---------------------------------  43
B  PF3D7_0937000   KSKNNEVYKKYNFDDLIYSLNSNDILKIIFPSHIFDKLYYELKDCDDNYNTYYLCEN-----------------------  79
B  PF3D7_0532400   --------------CERFIYYNPNVQGVYPDMFIASSSQTYSDGSEYDVMKYAN--------------------------  40
B  PF3D7_0532300   --CEKKPLNVDMKYVKSKCCDSISESNMVDRLKVQMLLKNAFETEDGNVFEQNH--------------------------  52
B  PF3D7_0401800   CSSNTRGFGCDCKDAANYILKNLDLYDEHEAKKHASDRTKTFSKELSKLFKKLRTKDPNMFNKNK---------------  126
B  PF3D7_1476300   --YENNNVDKHLNFSVSLENVNDLANSTKKEIEPKLQEILTNNDNNFNLLKKYES-------------------------  53
   PF3D7_0936600   --SELVDSKSGCLRSGENETNVKSSSSQTDSVLSSNNLTDFYNSYN----------------------------------  44
   PF3D7_1201200   -----ADVSISDIPIVKLHNSFDNVKEEFESENLEYEQHNESYYKHSPLED-----------------------------  46
   PF3D7_0731100   VPNDYKGRKKRNEDEDYMRESMYYVNSDSRGNNLTKEDEELLKSFYNNFNSSSNDFRKREEKFN----------------  121
B  PF3D7_1476200   KNSHIKNNTQQEEKNKNTTNQKSDANNKIHENKKKVEEEEKHKKEKNPNEKENN--------------------------  119
   PF3D7_0219700   LQHHHVKNITSQPLSSDLNDYSTEDEEVMNILSNNMTHNNNLTSNNSNVNQVEN--------------------------  57
   PF3D7_0219800   GYLSYKEKKIGSQSRNKRINSKNNNDSNKNKKNDYQIVGIKQNKGKKYNKKGNN--------------------------  94
   PF3D7_1016600   ---SEYQCIENYNFEEKDENFSNDDIFKNDEKSEYNYSGNVVEDFNFYMDKMNETDD-----------------------  54
   PF3D7_1200900   FGDYFNFEDDDSFDDKNPFDEANYFKHECDFDKYEKSTDSKINVEDKDNNIDDKNS------------------------  118
   PF3D7_1001700   --CELCIDNYQTLRNKNSEHKENDSHQQKDVNNEERKKKKKSKKYQDGKIDDFSN-------------------------  53
   PF3D7_1016500   MNKYFKNNKQKNLGSSYHSQFLDDYDDYPDEKIFANIPKKKNQNRNNISSKKMN--------------------------  117
   PF3D7_1016700   KYNDYDENCYETEGETKGSLYSDNYNKKKKTDSMNKIAKKDSCSDLQYDKKKEENK------------------------  118
   PF3D7_0936800   ----------AEASPEEHNNLRSHSTSDPKKNEEKSLSDEINKCDMKK--------------------------------  38
   PF3D7_0801000   FRNMYDRLRDEDENDYDQPPSTSSSNRGRGNERYSQSRDRREERNNYNSDYYTRGN------------------------  118
   PF3D7_0202100   ----AEGYMKGSGSEKNVYLSNKNKEINMNQQSDNKMCDECDDMNQPGDVNKND--------------------------  50
   PF3D7_1016800   ILKNLEKYIEENYGNCVEERCYYLKEPKVNNPYECEEKKKCLREEFEKCEPCQN--------------------------  57
   PF3D7_0830600   VLTEEDVQNINSSYNNEELNNNNNNNIRSTSENNVNTYELPNGELHNNSDRLNG--------------------------  119
   PF3D7_1148700   ------ASAMQHSLRGDFVTQRSTCFRTEKERKQKALNLIKNENNAEGYFNNLN--------------------------  48
   PF3D7_0424000   RNNDPEEQNKLEEERKKGEEEQNKLEEERKKREEEKSNDTFLSNNDTNSNGSTN--------------------------  119
   PF3D7_0532200   --SNVIEGFRNTFSNHEKKNKMKAHSCRTLNCTEQGIKPHNLRRAAK---------------------------------  45
   PF3D7_1001800   ---SDNNAKKEGRSGIEKVKSKSQHSPCSISNNAAVGYYDYLKIYNDG--------------------------------  45
   PF3D7_1201000_2 VLQEFVEGKISEEEDKQKYAENLDDDDEDDEEDDDDEDDEDNDDDEDDDDDDDED-------------------------  115
   PF3D7_1477400   -NDIIHMGLENDSESDEFKSIIEDNTIVVEDSTDTSYKDARYFYTEHIFSSYTS--------------------------  53
   PF3D7_1477300   ELVLANYPSSNYAFSNYASANYSSSNYPSSKMDDLKRHENNCSSSNKAVD------------------------------  51
```

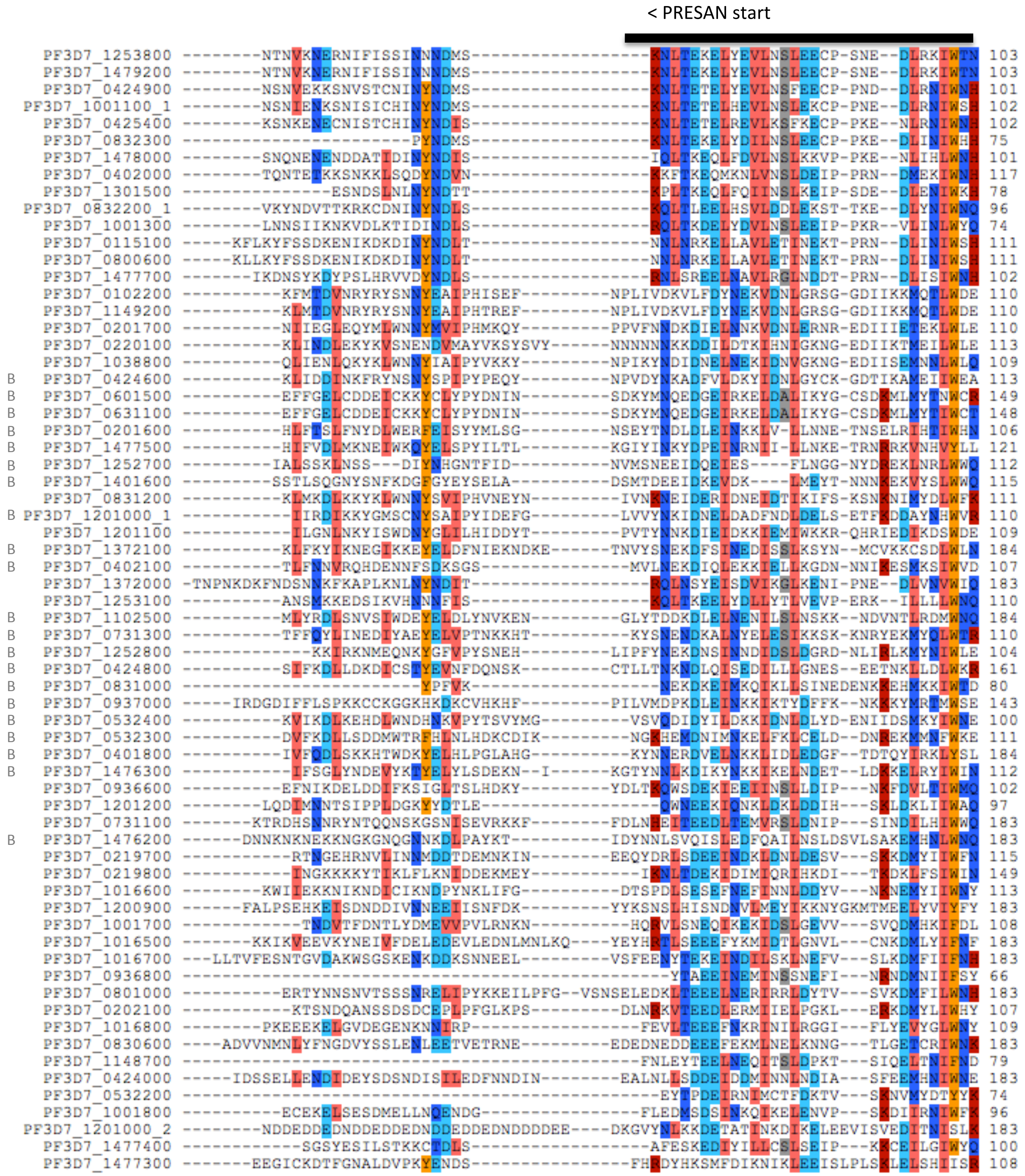

< PRESAN start
PF3D7_1253800 --------NTNVKNERNIFISSINNNDMS-------------------KNLTEKELYEVLNSLEECP-SNE--DLRKIWTN 103
PF3D7_1479200 --------NTNVKNERNIFISSINNNDMS-------------------KNLTEKELYEVLNSLEECP-SNE--DLRKIWTN 103
PF3D7_0424900 --------NSNVEKKSNVSTCNINYNDMS-------------------KNLTETELYEVLNSFEECP-PND--DLRNIWNH 101
PF3D7_1001100_1 --------NSNIENKSNISICHINYNDMS-------------------KNLTETELHEVLNSLEKCP-PNE--DLRNIWSH 102
PF3D7_0425400 --------KSNKENECNISTCHINYNDIS-------------------KNLTETELREVLKSFKECP-PKE--NLRNIWNH 102
PF3D7_0832300 ----------------------PYNDMS-------------------KNLTEKELYDIILNSLEECP-PKE--DLINIWHH 75
PF3D7_1478000 --------SNQNENENDDATIDINYNDIS-------------------IQLTKEQLFDVLNSLKKVP-PKE--NLIHLWNH 101
PF3D7_0402000 --------TQNTETKKSNKKLSQDYNDVN-------------------KKFTKEQMKNLVNSLDEIP-PRN--DMEKIWNH 117
PF3D7_1301500 ---------------ESNDSLNLNYNDTT-------------------KPLTKEQLFQIINSLKEIP-SDE--DLENIWKH 78
PF3D7_0832200_1 --------VKYNDVTTKRKCDNINYNDLS-------------------KQLTLEELHSVLDDLEKST-TKE--DLYNIWNQ 96
PF3D7_1001300 --------LNNSIIKNKVDLKTIDINDLS-------------------RQLTKDELYDVLNSLEEIP-PKR--VLINLWYQ 74
PF3D7_0115100 -----KFLKYFSSDKENIKDKDINYNDLT-------------------NNLNRKELLAVLETINEKT-PRN--DLINIWSH 111
PF3D7_0800600 -----KLLKYFSSDKENIKDKDINYNDLT-------------------NNLNRKELLAVLETINEKT-PRN--DLINIWSH 111
PF3D7_1477700 -------IKDNSYKDYPSLHRVVDYNDLS-------------------RNLSREELNAVLRGLNDDT-PRN--DLISIWNH 102
PF3D7_0102200 ----------KFMTDVNRYRYSNNYEAIPHISEF---------NPLIVDKVLFDYNEKVDNLGRSG-GDIIKKMQTLWDE 110
PF3D7_1149200 ----------KLMTDVNRYRYSNNYEAIPHTREF---------NPLIVDKVLFDYNEKVDNLGRSG-GDIIKKMQTLWDE 110
PF3D7_0201700 ----------NIIEGLEQYMLWNNYMVIPHMKQY---------PPVFNNDKDIELNNKVDNLERNR-EDIIIETEKLWLE 110
PF3D7_0220100 ----------KLINDLEKYKVSNENDVMAYVKSYSVY------NNNNNNKKDDILDTKIHNIGKNG-EDIIKTMEILWLE 113
PF3D7_1038800 ----------QLIENLQKYKLWNNYIAIPYVKKY---------NPIKYNDIDNELNEKIDNVGKNG-EDIISEMNNLWLQ 109
B PF3D7_0424600 ----------KLIDDINKFRYNSNYSPIPYPEQY---------NPVDYNKADFVLDKYIDNLGYCK-GDTIKAMEIIWEA 113
B PF3D7_0601500 ----------EFFGELCDDEICKKYCLYPYDNIN---------SDKYMNQEDGEIRKELDALIKYG-CSDKMLMYTNWCR 149
B PF3D7_0631100 ----------EFFGELCDDEICKKYCLYPYDNIN---------SDKYMNQEDGEIRKELDALIKYG-CSDKMLMYTIWCT 148
B PF3D7_0201600 ----------HLFTSLFNYDLWERFEISYYMLSG---------NSEYTNDLDLEINKKLV-LLNNE-TNSELRIHTIWHN 106
B PF3D7_1477500 ----------HIFVDLMKNEIWKQYELSPYILTL---------KGIYINKYDPEINRNII-LLNKE-TRNRRKVNHVYLL 121
B PF3D7_1252700 ---------IALSSKLNSS---DIYNHGNTFID----------NVMSNEEIDQEIES----FLNGG-NYDRKKLNRLWWQ 112
B PF3D7_1401600 ---------SSTLSQGNYSNFKDGFGYEYSELA----------DSMTDEEIDKEVDK----LMEYT-NNNKEKVYSLWWQ 115
PF3D7_0831200 ----------KLMKDLKKYKLWNNYSVIPHVNEYN--------IVNKNEIDERIDNEIDTIKIFS-KSNKNIMYDLWFK 111
B PF3D7_1201000_1 ----------IIRDIKKYGMSCNYSAIPYIDEFG--------LVVYNKIDNELDADFNDLDELS-ETFKDDAYNHWVR 110
PF3D7_1201100 ----------ILGNLNKYISWDNYGLILHIDDYT--------PVTYNNKDIEIDKKIEMIWKKR-QHRIEDIKDSWDE 109
B PF3D7_1372100 ----------KLFKYIKNEGIKKEYELDFNIEKNDKE------TNVYSNEKDFSINEDISSLKSYN--MCVKKCSDLWLN 184
B PF3D7_0402100 ----------TLFNNVRQHDENNFSDKSGS---------------MVLNEKDIQLEKKIELLKGDN-NNIKESMKSIWVD 107
PF3D7_1372000 -TNPNKDKFNDSNNKFKAPLKNLNYNDIT-------------------RQLNSYEISDVIKGLKENI-PNE--DLVNVWIQ 183
PF3D7_1253100 ----------ANSMKKEDSIKVHNNNFIS-------------------KQLTKEELYDLLYTLVEVP-ERK--ILLLLWNQ 110
B PF3D7_1102500 ----------MLYRDLSNVSIWDEYELDLYNVKEN---------GLYTDDKDLELNENILSLNSKK--NDVNTLRDMWNQ 184
B PF3D7_0731300 ----------TFFQYLINEDIYAEYELVPTNKKHT----------KYSNENDKALNYELESIKKSK-KNRYEKMYQLWTR 110
B PF3D7_1252800 -------------KKIRKNMEQNKYGFVPYSNEH---------LIPFYNEKDNSINNDIDSLDGRD-NLIRLKMYNIWLE 104
B PF3D7_0424800 ----------SIFKDLLDKDICSTYEVNFDQNSK---------CTLLTNKNDLQISEDILLLGNES-EETNKLLDLWKR 161
B PF3D7_0831000 ----------------------YPFVK--------------------NEKDKEIMKQIKLLSINEDENKKEHMKKIWTD 80
B PF3D7_0937000 -----IRDGDIFFLSPKKCCKGGKHKDKCVHKHF---------PILVMDPKDLEINKKIKTYDFFK--NKKKYMRTMWSE 143
B PF3D7_0532400 ----------KVIKDLKEHDLWNDHNKVPYTSVYMG--------VSVQDIDYILDKKIDNLDLYD-ENIIDSMKYIWNE 100
B PF3D7_0532300 ----------DVFKDLLSDDMWTRFHLNLHDKCDIK--------NGKHEMDNIMNKELFKLCELD--DNREKMMNFWKE 111
B PF3D7_0401800 ----------IVFQDLSKKHTWDKYELHLPGLAHG---------KYNNERDVELNKKLIDLEDGF--TDTQYIRKLYSL 184
B PF3D7_1476300 ----------IFSGLYNDEVYKTYELYLSDEKN--I------KGTYNNLKDIKYNKKIKELNDET--LDKKELRYIWIN 112
PF3D7_0936600 ----------EFNIKDELDDIFKSIGLTSLHDKY---------YDLTKQWSDEKIEEIINSLLDIP---NKFDVLTIWMQ 102
PF3D7_1201200 --------LQDIMNNTSIPPLDGKYYDTLE-------------------QWNEEKIQNKLDKLDDIH---SKLDKLIIWAQ 97
PF3D7_0731100 -------KTRDHSNNRYNTQQNSKGSNISEVRKKF---------FDLNHEITEEDLTEMVRSLDNIP---SINDILHIWWQ 183
B PF3D7_1476200 ------DNNKNKNEKKNGKGNQGNNKDLPAYKT-----------IDYNNLSVQISLEDFQAILNSLDSVLSAKEMHNLWNQ 183
PF3D7_0219700 ----------RTNGEHRNVLINNMDDTDEMNKIN---------EEQYDRLSDEEINDKLDNLDESV---SKKDMYIIWFN 115
PF3D7_0219800 ----------INGKKKKYTIKLFLKNIDDEKMEY--------------IKNLTDEKIDIMIQRIHKDI---TKDKLFSIWIN 149
PF3D7_1016600 ----------KWIIEKKNIKNDICIKNDPYNKLIFG---------DTSPDLSESEFNEFINNLDDYV---NKNEMYIIWNY 113
PF3D7_1200900 ------FALPSEHKEISDNDDIVNNEEIISNFDK----------YYKSNSLHISNDNVLMEYIKKNYGKMTMEELYVIYFY 183
PF3D7_1001700 ------------TNDVTFDNTLYDMEVVPVLRNKN----------HQRVLSNEQIKEKIDSLGEVV---SVQDMHKIFDL 108
PF3D7_1016500 -------KKIKVEEVKYNEIVFDELEDEVLEDNLMNLKQ----YEYHRTLSEEEFYKMIDTLGNVL---CNKDMLYIFNF 183
PF3D7_1016700 ---LLTVFESNTGVDAKWSGGSKENKDDKSNNEEL---------VSFEENYTEKEINDILSKLNEFV---SLKDMFIIFNH 183
PF3D7_0936800 --YTAEEINEMINSSNEFI---NRNDMNIIFSY 66
PF3D7_0801000 ----------ERTYNNSNVTSSSNRELIPYKKEILPFG--VSNSELEDKLTEEELNERIRRLDYTV---SVKDMFILWNH 183
PF3D7_0202100 -----------KTSNDQANSSDSDCEPLPFGLKPS----------DLNRKVTEEDLERMIIELPGKL---ERKDMYLIWHY 107
PF3D7_1016800 ---------PKEEEKELGVDEGENKNNIRP-----------------FEVLTEEEFNKRINILRGGI---FLYEVYGLWNY 109
PF3D7_0830600 ----ADVVNMNLYFNGDVYSSLENLEETVETRNE----------EDEDNEDDEEEFEKMLNELKNNG---TLGETCRIWNK 183
PF3D7_1148700 ---FNLEYTEELNEQITSLDPKT---SIQELTNIFND 79
PF3D7_0424000 -----IDSSELLENDIDEYSDSNDISILEDFNNDIN---------EALNLLSDDEIDDMINNLNDIA---SFEEMHNIWNE 183
PF3D7_0532200 ---EYTPDEIRNIMCTFDKTV---SKNVMYDTYYK 74
PF3D7_1001800 ----------ECEKELSESDMELLNQENDG------------------FLEDMSDSINKQIKELENVP---SKDIIRNIWFK 96
PF3D7_1201000_2 --------NDDEDDEDNDDEDDEDNDDEDDEDNDDDDEE----DKGVYNLKKDETATINKDIKELEEVISVEDITNISLK 183
PF3D7_1477400 -------------SGSYESILSTKKCTDLS-------------------AFESKEDIYILLCSLSEIP---KKCEILGIWYQ 100
PF3D7_1477300 -------EEGICKDTFGNALDVPKYENDS-------------------FHRDYHKSMFDIKNIKLEEISLPLSKLELSHIISR 108

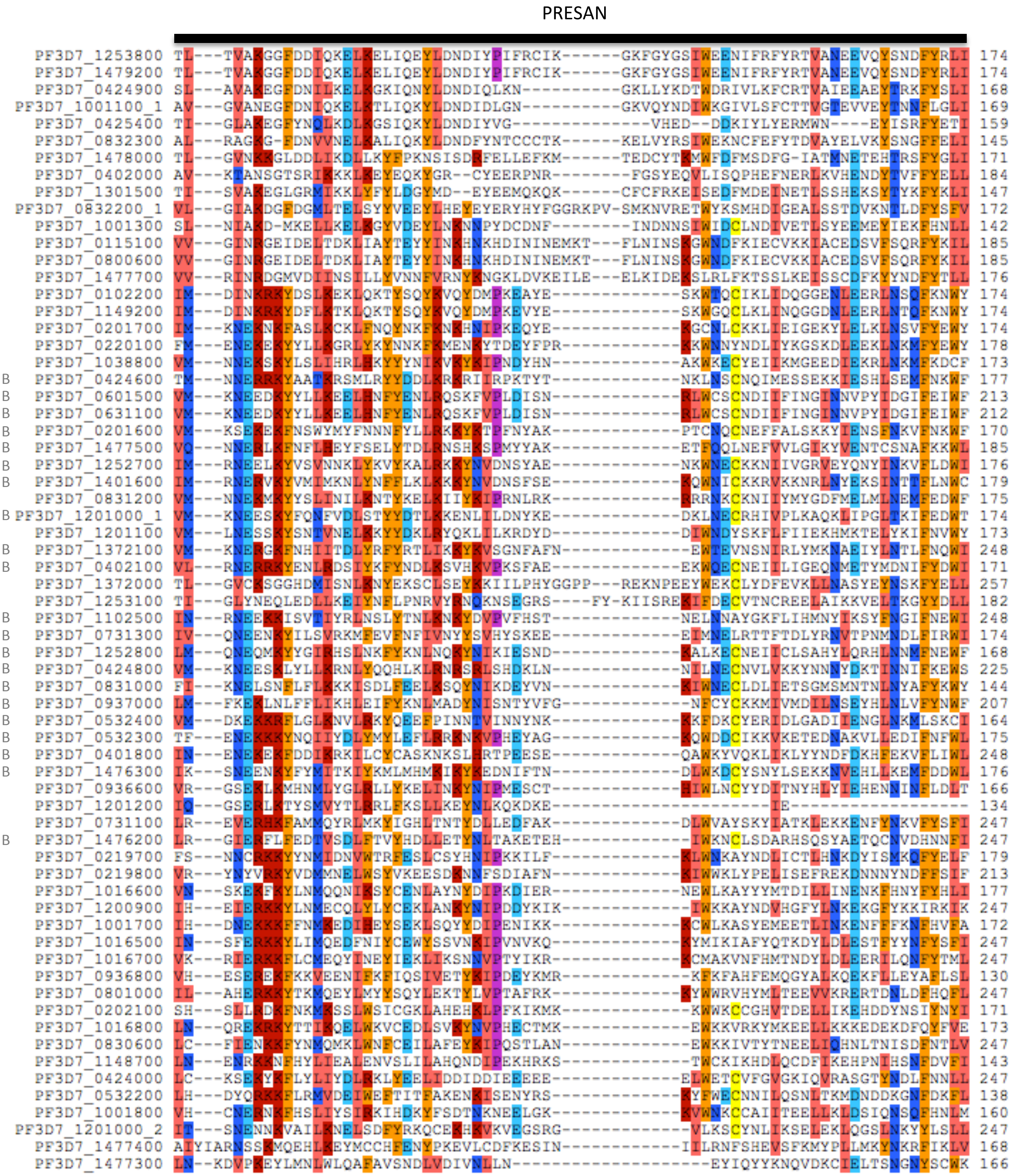
PRESAN
PF3D7_1253800 174
PF3D7_1479200 174
PF3D7_0424900 168
PF3D7_1001100_1 169
PF3D7_0425400 159
PF3D7_0832300 145
PF3D7_1478000 171
PF3D7_0402000 184
PF3D7_1301500 147
PF3D7_0832200_1 172
PF3D7_1001300 142
PF3D7_0115100 185
PF3D7_0800600 185
PF3D7_1477700 176
PF3D7_0102200 174
PF3D7_1149200 174
PF3D7_0201700 174
PF3D7_0220100 178
PF3D7_1038800 173
B PF3D7_0424600 177
B PF3D7_0601500 213
B PF3D7_0631100 212
B PF3D7_0201600 170
B PF3D7_1477500 185
B PF3D7_1252700 176
B PF3D7_1401600 179
PF3D7_0831200 175
B PF3D7_1201000_1 174
PF3D7_1201100 173
B PF3D7_1372100 248
B PF3D7_0402100 171
PF3D7_1372000 257
PF3D7_1253100 182
B PF3D7_1102500 248
B PF3D7_0731300 174
B PF3D7_1252800 168
B PF3D7_0424800 225
B PF3D7_0831000 144
B PF3D7_0937000 207
B PF3D7_0532400 164
B PF3D7_0532300 175
B PF3D7_0401800 248
B PF3D7_1476300 176
PF3D7_0936600 166
PF3D7_1201200 134
PF3D7_0731100 247
B PF3D7_1476200 247
PF3D7_0219700 179
PF3D7_0219800 213
PF3D7_1016600 177
PF3D7_1200900 247
PF3D7_1001700 172
PF3D7_1016500 247
PF3D7_1016700 247
PF3D7_0936800 130
PF3D7_0801000 247
PF3D7_0202100 171
PF3D7_1016800 173
PF3D7_0830600 247
PF3D7_1148700 143
PF3D7_0424000 247
PF3D7_0532200 138
PF3D7_1001800 160
PF3D7_1201000_2 247
PF3D7_1477400 168
PF3D7_1477300 166

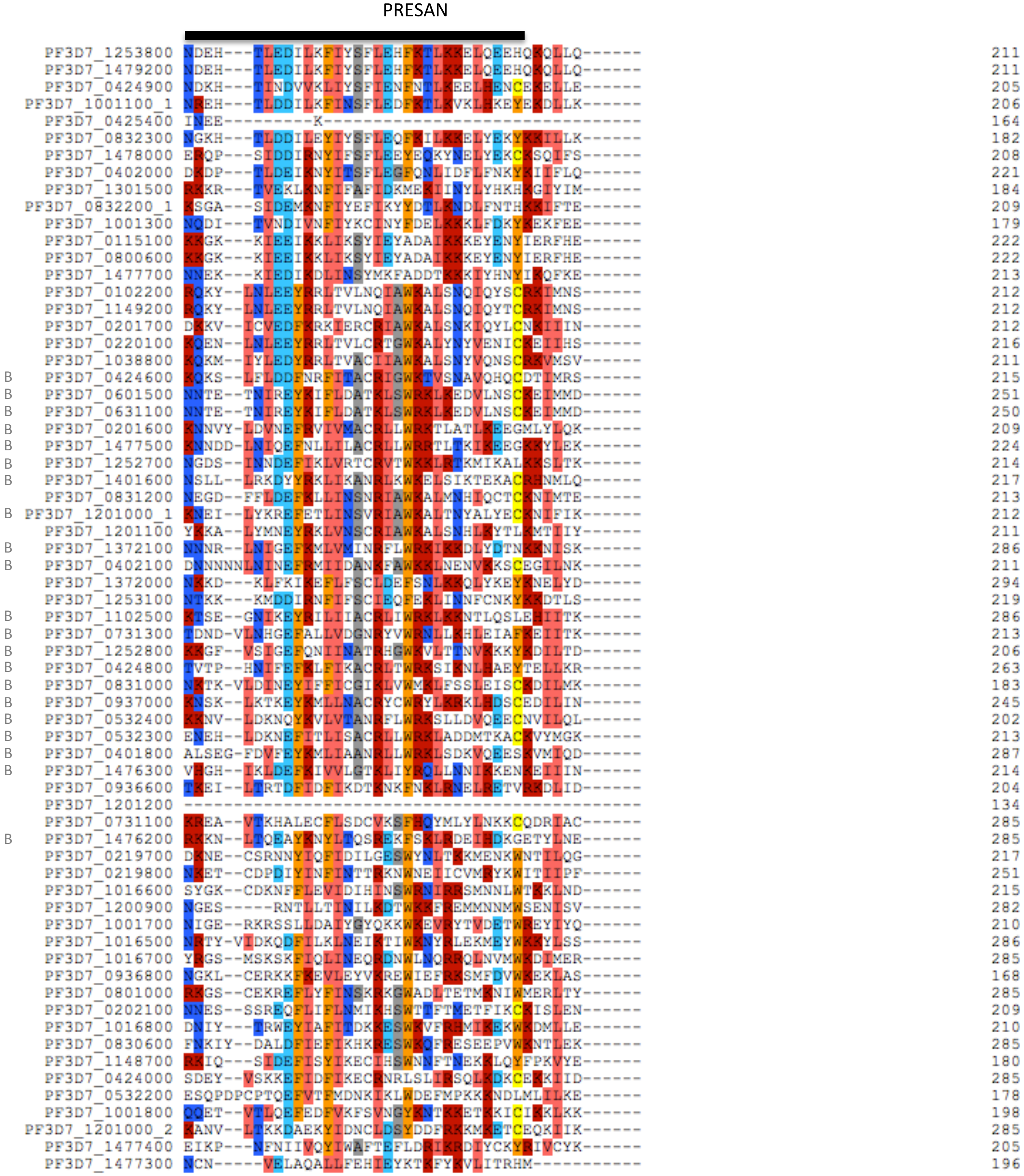

Supplementary figure 4

ClustalX alignment of the extended PRESAN domains of *P. falciparum* PHIST proteins (annotated in PlasmoDB). Sequences included 150 residues N-terminal to the annotated PRESAN domain (or to the cleaved HT motif, whichever was shorter) and six residues C-terminal to the annotated PRESAN domain. The N-terminal end of the PRESAN domain (Pfam ID: PF09687) in PHISTb protein PF3D7_0201600 is annotated by a black bar above the aligned sequences. Residue positions with greater than 30% amino acid identity/similarity are coloured (alignment was coloured using Multiple Align Show). Proteins annotated as PHISTb family members in plasmoDB are indicated by a letter 'B'.
