# Supplementary material for: A conserved domain targets exported PHISTb family proteins to the periphery of Plasmodium infected erythrocytes
Source: Mol Biochem Parasitol. 2014 Aug;196(1):29–40. doi: 10.1016/j.molbiopara.2014.07.011 (PMC4165601; doi:10.1016/j.molbiopara.2014.07.011)
Supplement: Supplementary file 5 [file mmc5.docx]

**Supplementary methods**

**Quantitation of fluorescence intensity at the erythrocyte plasma membrane.**

Using Image J, a line of 40 pixels (3.9 um) in length was drawn on deconvolved fluorescence images of infected erythrocytes (images were 170 x 170 pixels). As indicated in supplementary figure 1, the start of the line was placed outside of the infected cell, the centre point of the line was placed approximately at the plasma membrane of the infected erythrocyte, and the end of the line was placed within the erythrocyte cytoplasm. The line was positioned such that it was approximately perpendicular to the infected erythrocyte plasma membrane, and did not extend into the parasite. Pixel intensity values along the line were determined. Background fluorescence was calculated as the mean fluorescence of pixels 1 – 5. The membrane was determined as the pixel with maximal fluorescence intensity between pixels 15 and 25. Erythrocyte cytosolic fluorescence was calculated as the mean fluorescence intensity between pixels 30 and 35. Background fluorescence was deducted from both erythrocyte plasma membrane peak fluorescence and erythrocyte cytosolic fluorescence. Fold difference at the membrane was determined as an average ratio of background-corrected membrane peak fluorescence:background-corrected erythrocyte cytosolic fluorescence for 10 infected erythrocytes.
